# Supplementary material for: Semantic regularization of electromagnetic inverse problems
Source: Nat Commun. 2024 May 8;15:3869. doi: 10.1038/s41467-024-48115-5 (PMC11079068; doi:10.1038/s41467-024-48115-5)
Supplement: Supplementary file 1 — Supplementary Information [file 41467_2024_48115_MOESM1_ESM.pdf]

Supplementary Information for

Semantic regularization of electromagnetic inverse problems

Hongrui Zhang[1+], Yanjin Chen[1+], Zhuo Wang[1], Tie Jun Cui[2,3], Philipp del Hougne[4] and Lianlin Li[1,3]

[1] State Key Laboratory of Advanced Optical Communication Systems and Networks,

School of Electronics, Peking University, Beijing 100871, China

[2] State Key Laboratory of Millimeter Waves, Southeast University, Nanjing 210096, China

[3] Pazhou Laboratory (Huangpu), Guangzhou, Guangdong 510555, China

[4] Univ Rennes, CNRS, IETR - UMR 6164, F-35000 Rennes, France

[+] These authors contributed equally to this work.

Outline:

Supplementary Note 1. Details of proposed networks and training algorithms

Supplementary Note 2. Comparison of proposed semantic regularization with existing data-driven regularizations

Supplementary Note 3. Setups of inverse-scattering experiments

Supplementary Note 4. Supplementary inverse-scattering results for Fig. 2e

Supplementary Note 5. Noise immunity experiments

Supplementary Note 6. Comparison of semantic regularization with other regularizations such as L1, L2 and TV

Supplementary Note 7. Generalization experiment for EM inverse problem

Supplementary Note 8. Evaluation of the semantic regularization with different influence factors

Supplementary Note 9. System configuration of microwave metasurface camera and details of microwave compressive imaging experiments

Supplementary Note 10. Details of 4D compressive microwave meta-imaging experiments

Supplementary Note 11. Supplementary results for 4D compressive microwave meta-imaging experiments

Supplementary Note 12. Integration of semantic regularization with iterative inverse-scattering approaches

Supplementary Note 13. MSE statistics of obtained solutions

Supplementary Note 14. Applying the proposed semantic regularization in other domains (reservoir fluid)

Supplementary Note 1. Details of proposed networks and training algorithms

The internal structure of the network in **Fig. 1b** is depicted in detail in **Supplementary Figure 1.1(a)**, which contains the following five modules: encoder, decoder, pre-trained large language model (LLM, BERT here), discriminator and semantic converter. The five modules have variable structures for different forms of data. **Supplementary Figure 1.2** shows the structure of the modules for 2D images; while **Supplementary Figure 1.3** shows the structure of the modules for 4D skeletons. Below we will introduce the detailed structures and functions of different modules respectively.

(a)

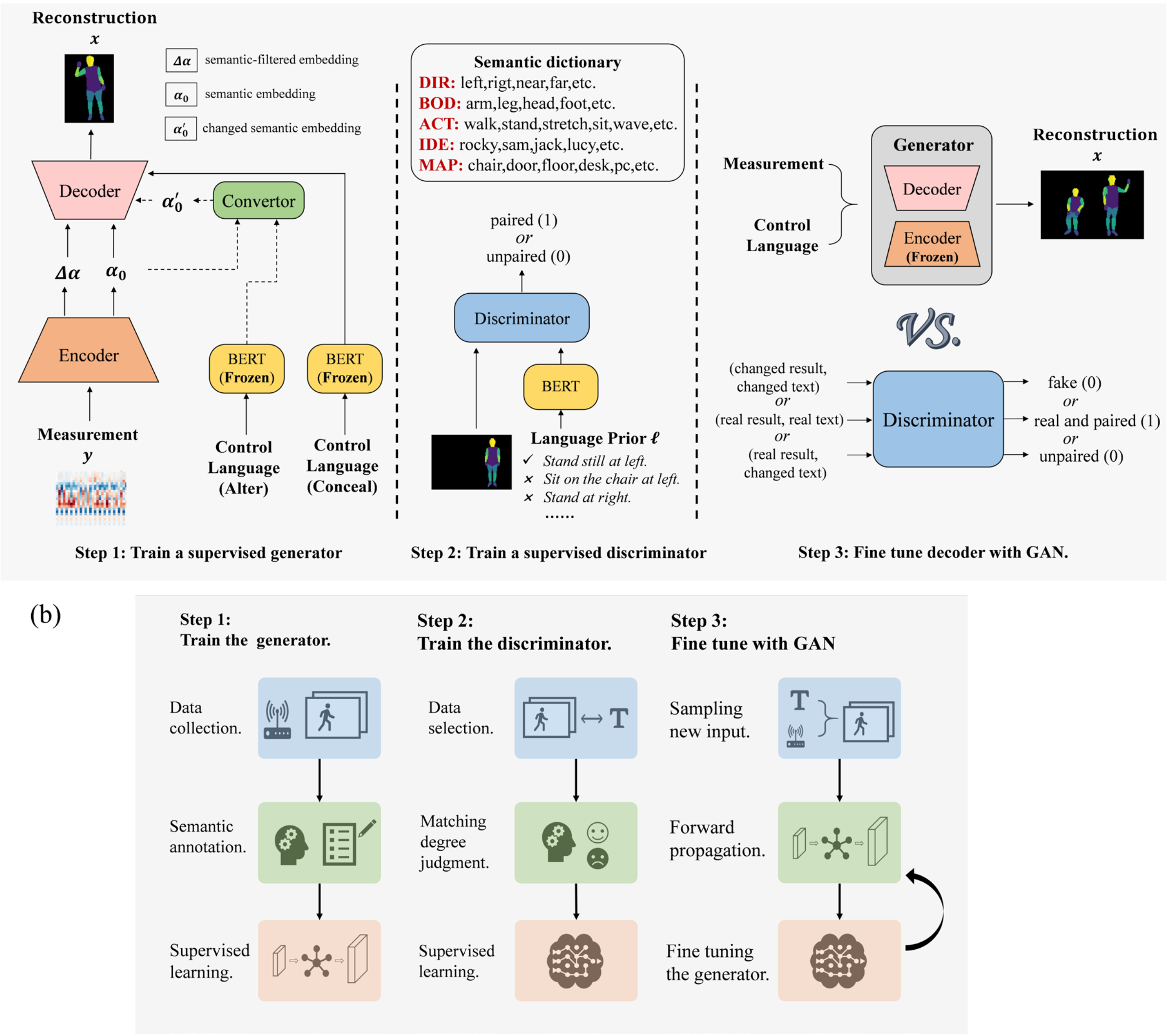

Supplementary Figure 1.1 | Designed networks and training process. (a) The overall network structure. **(b)** The complete training pipeline of our model which is divided into three main steps.

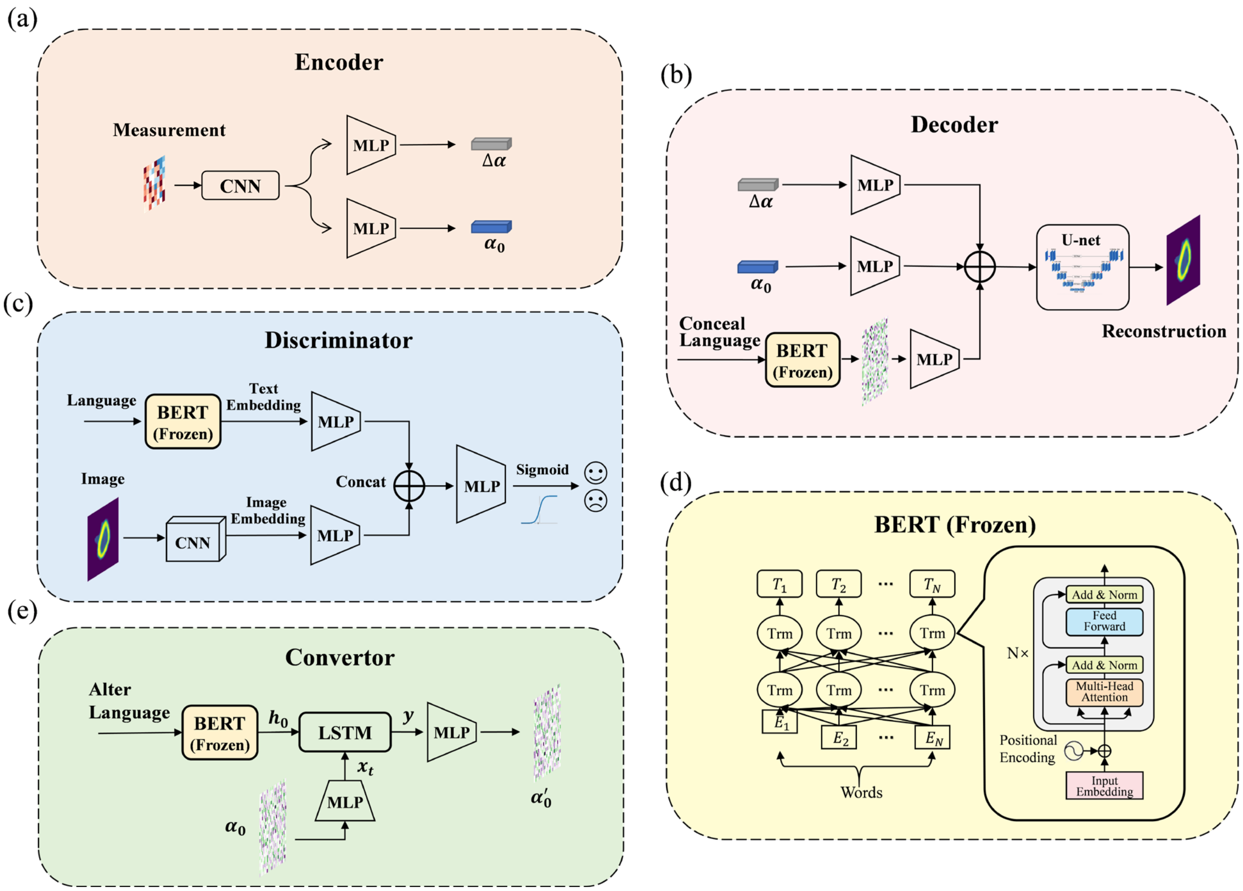

Supplementary Figure 1.2 | Internal composition of the network modules for 2D reconstruction. (a) The architecture of the 'Encoder'. **(b)** The architecture of the 'Decoder'. **(c)** The architecture of the 'Discriminator'. **(d)** The architecture of the LLM 'BERT'. **(e)** The architecture of the 'Convertor'. Here, MLP is the multilayer perceptron which is composed by cascaded fully connected neurons and activation functions (ReLU)[1], CNN represents 2D convolutional neural network, 'Trm' is short for the transformer block.

The pre-trained LLM 'BERT' play a key role in the proposed network, converting the input language prior ℓ into the corresponding semantic embedding vector α_0, which can be modeled as $\alpha_0 = Bert(\ell)$. **Supplementary Figure 1.2(d)** is the architecture of the BERT we use. It is a transformers[2] model pretrained on a large corpus of English data in a self-supervised fashion. This means it was pretrained on the raw texts only, with no humans labeling them. The model learns an inner representation of the English language that can then be used to extract features useful for downstream tasks. In this paper the pre-trained BERT is frozen and used directly for text embedding extraction. Specifically, the pre-trained BERT is 'bert-base-uncased' from Hugging Face[3], which has 110M parameters. It was pretrained on BookCorpus, a dataset consisting of

11,038 unpublished books and English Wikipedia (excluding lists, tables and headers). The model was trained on 4 cloud TPUs in Pod configuration (16 TPU chips total) for one million steps with a batch size of 256. The sequence length was limited to 128 tokens for 90% of the steps and 512 for the remaining 10%. The optimizer used is Adam with a learning rate of 1×10^{-4}, $\beta_1 = 0.9$ and $\beta_2 = 0.999$, a weight decay of 0.01, learning rate warmup for 10,000 steps and linear decay of the learning rate after. BERT inserts a [CLS] token at the beginning of the sentence to indicate the semantics of the entire sentence.

Here, we would like to clarify the concept of 'Embedding', which plays a crucial role in exploiting semantic regularization. The term 'Embedding' refers to the vector representation obtained by mapping data through a trained neural network into a low-dimensional real-number space. This process allows the neural network to learn meaningful representations of the input data in a more compact form, facilitating various downstream tasks. Here, we directly use the pre-trained LLM BERT to transform the text into the corresponding embedding vector, and align the obtained semantic embedding vector from the encoder network under corresponding microwave measurement with it. This allows the computer to better understand the similarities and associations between words, and makes semantic regularization have strong generalization and zero-shot capability. Specifically, the pre-trained BERT transforms each input word into a corresponding embedding vector of length 768. The complete input corresponds to an embedding vector of dimensions n×768, where n increases with the length of the input sentence, and we can restrict the length n to a fixed value by filling and truncating in practice. The following **Supplementary Figure 1.3** illustrates the embeddings corresponding to three different input texts, labeled as 'a', 'an', and 'two'. Here, we just take the first dimension ([CLS]) and reshape it from 1×768 to a 24×32 vector. One thing of notice is that the embeddings for input 'a' and 'an' are notably more similar than 'two', which is consistent with common sense and indicates that the pre-trained BERT can automatically capture semantic relationships between different input texts, thus achieving strong zero-shot capability.

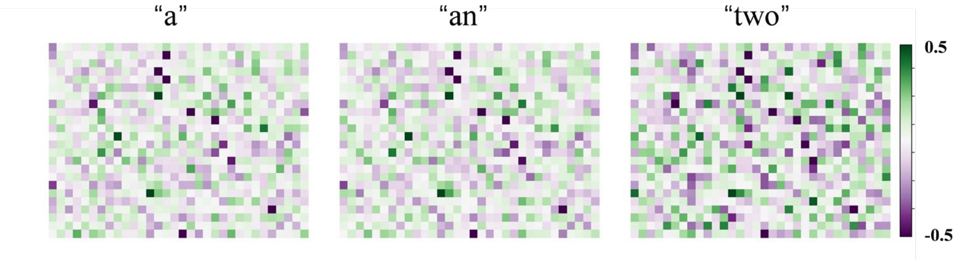

Supplementary Figure 1.3 | Embeddings corresponding to different texts.

Supplementary Figure 1.2(a) is the architecture of the 'Encoder'. It consists of the convolutional neural network (CNN)[4-6] and the multilayer perceptron (MLP)[7,8]. The input to the encoder is microwave measurement, and the outputs are the semantic embedding α_0 and the semantic-filtered embedding $\Delta\alpha$. The microwave is processed by CNN and MLP to obtain the microwave feature. Then, the microwave feature is processed by two MLPs to obtain the semantic-filtered embedding $\Delta\alpha$ and the semantic embedding α_0.

Supplementary Figure 1.2(b) is the architecture of the 'Decoder'. It consists of the MLP and the U-net[5]. The inputs to the decoder are the semantic embedding α_0, the semantic-filtered embedding $\Delta\alpha$, and the output is the reconstruction result (i.e., 2D image). In the actual experiment, we introduce an optional input port to the decoder, specifically designed for generating concealed reconstructions in the context of privacy preservation. This means that we can directly input the control language (conceal) corresponding to what we wish to conceal. Then the control embedding is extracted from the control language (conceal) by the frozen BERT. This additional input is then processed by BERT and used as input to the decoder, allowing us to directly conceal a portion or the entirety of the reconstruction without altering the original semantic embedding α_0, achieving effective privacy protection. The feature of the semantic-filtered embedding $\Delta\alpha$ is extracted by MLP, the feature of the semantic embedding α_0 is extracted by another MLP, and the control embedding is processed by MLP to obtain the control feature. These three features are concatenated together to integrate the information, and then fed into a U-net to obtain the reconstruction result.

Supplementary Figure 1.2(c) is the architecture of the 'Discriminator'. It is a pre-trained network consisting of the CNN and MLP. The inputs of the discriminator are the semantic and the image, and the output is '0' or '1', indicating whether the image matches the semantic or whether the image is realistic and meaningful. Output '0' indicates that the semantic does not match the image

(unpaired), such as the image does not match the description of the semantic itself, the image is faked, or the quality of the image does not meet the requirement. Output '1' indicates that the semantic and image are artificially judged to be paired, that is, the reconstruction is sufficient to match the semantic. Here the text embedding is also extracted from the semantic by the frozen BERT, and then fed into the MLP to obtain the semantic feature. The CNN extracts the image embedding from the input image and this embedding is fed into the MLP to obtain the image feature. These two features are concatenated together and fed into the MLP, and finally the discriminative result is output after a Sigmoid activation function.

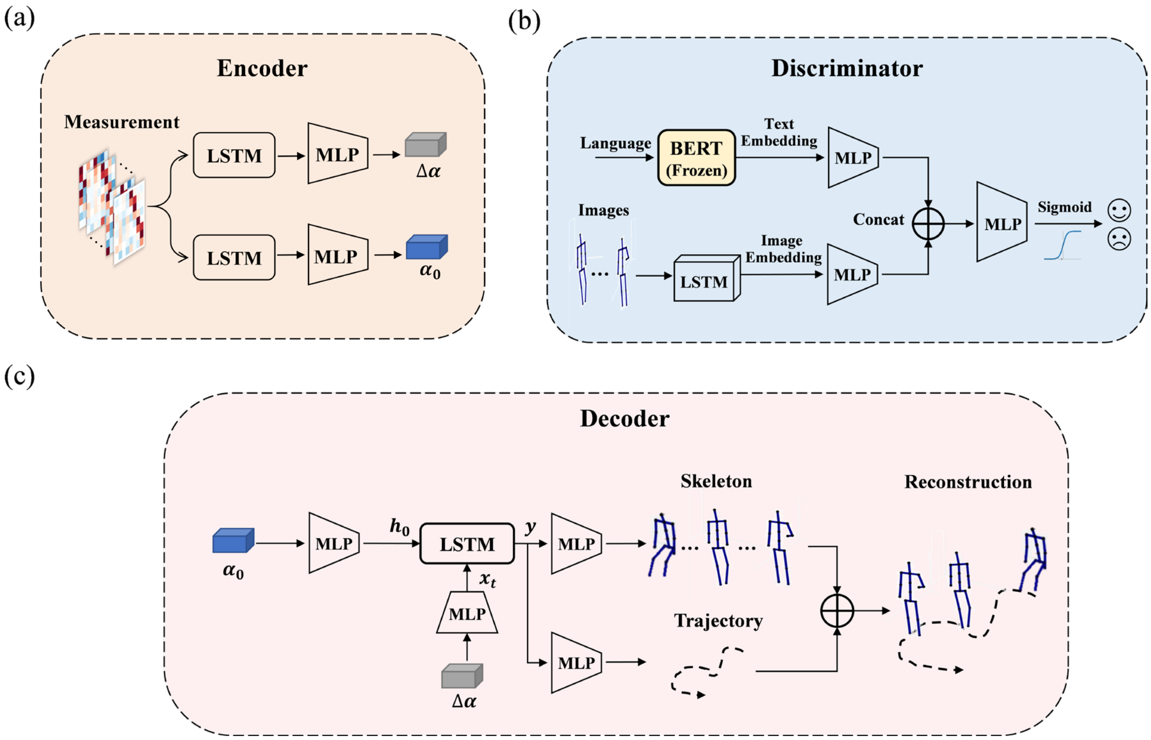

Supplementary Figure 1.4 | Internal composition of the network modules for 4D skeleton key-points. (a) The architecture of the 'Encoder'. **(b)** The architecture of the 'Discriminator'. **(c)** The architecture of the 'Decoder'.

For the 4D skeleton key-points imaging experiment, the structures of the modules 'BERT' and 'Convertor' are the same as in **Supplementary Figure 1.2(d)(e)**. The structures of other modules need to be adjusted because the input and output are in the form of a sequence. **Supplementary Figure 1.4(a)** shows the architecture of the 'Encoder'. The input microwave sequence (consisting of 25 frames within 1.5s in our experiment) is transformed by LSTM and MLP into semantic-filtered embedding $\Delta\alpha$ and semantic embedding α_0, respectively. **Supplementary Figure 1.4(b)**

is the architecture of the 'Discriminator'. The input is the semantic and corresponding skeleton sequence whose embedding feature is obtained through LSTM. **Supplementary Figure 1.4(c)** is the architecture of the 'Decoder'. The vector h_0 obtained after processing the semantic embedding α_0 by the MLP is used as the initial state of the LSTM, and the semantic-filtered embedding $\Delta\alpha$ processed by MLP is inputted to the LSTM to obtain the corresponding feature sequence. Through another two MLP, the skeleton sequence containing only actions without positional information and its trajectory are obtained respectively. The final complete reconstruction is obtained by adding the skeleton sequence of the action and the motion trajectory.

The training of the network is organized in three steps, as shown in **Supplementary Figure 1.1**. The first step is the supervised training of the generator using the collected triple dataset (including semantics, microwave measurements and objects), involving the training of the encoder and decoder. The specific loss function for this step is as follows

$$L_1 = \frac{1}{2}\sum_{i=1}^{M}(\|d(e(y_i)) - x_i\|_2^2 + \gamma\left\|\alpha_{0,i} - Bert(\ell_i)\right\|_2^2 + \|\Delta\alpha_i\|_2^2) \qquad \text{(S1.1)}$$

where $e(\cdot)$ and $d(\cdot)$ stand for the encoder and the decoder, and $(\Delta\alpha_i, \alpha_{0,i})$=$e(y_i)$ represent the semantic-filtered component and the semantic embedding generated by the encoder, respectively. $Bert(\cdot)$ donates the operation of the frozen BERT. y_i is the microwave measurement and x_i is the corresponding object to be reconstructed. ℓ_i is the true language prior which is consistent with the object x_i. The optimizer used is AdamW[9] with a learning rate of 1×10^{-3}, $\beta_1 = 0.9$ and β_2 = 0.99, batch size=100, epochs=1000. γ in the above equation is set as 1.0 because the three losses during training are of the same order of magnitude.

The second step is the supervised training of the discriminator using data pairs including objects and languages. The specific loss function for this step is as follows

$$L_2 = \frac{1}{2}\sum_{i=1}^{M}(-\log\big(dis(x_i, Bert(\ell_i))\big) - \log\big(1 - dis(x_i, Bert(\ell_i'))\big)) \qquad \text{(S1.2)}$$

where $dis(\cdot)$ stands for the discriminator. ℓ_i is the true language prior which is consistent with the object x_i, while ℓ_i' is another language prior that does not match x_i. The optimizer used is AdamW with a learning rate of 1×10^{-3}, $\beta_1 = 0.9$ and $\beta_2 = 0.99$, batch size=100, epochs=1000. In the third step, we freeze the network modules other than the decoder and discriminator and utilize GAN to fine tune them to further enhance the imaging results. The specific loss function for this step is as follows

$$L_{gen} = \frac{1}{2}\sum_{i=1}^{M}(\left\|d\left(e(y_i)\right) - x_i\right\|_2^2 - \log\left(dis\left(d\left(\Delta\alpha_i, \alpha'_{0,i}\right), Bert(\ell'_i)\right)\right) \quad \text{(S1.3)}$$

$$L_{dis} = \frac{1}{2}\sum_{i=1}^{M}(-\log\left(1 - dis\left(d\left(\Delta\alpha_i, \alpha'_{0,i}\right), Bert(\ell'_i)\right)\right) - \log\left(dis(x_i, Bert(\ell_i))\right) - \log\left(1 - dis(x_i, Bert(\ell'_i))\right)) \quad \text{(S1.4)}$$

where $\alpha'_{0,i}$, corresponds to the new language prior ℓ'_i, is the changed semantic embedding different from $\alpha_{0,i}$. The optimizer used is AdamW with a learning rate of 1×10^{-5}, $\beta_1 = 0.9$ and $\beta_2 = 0.99$, batch size=100, epochs=200.

Here we elaborate on how to alter the original semantic embedding α_0 to get the new semantic embedding α'_0. As one of the inputs of the decoder, the semantic embedding α_0 could be the output of the encoder based on the measurement. However, if we want to get solutions with other semantics under the same measurement, we can directly specify a new semantic embedding α'_0 to replace the original semantic embedding α_0 recommended from the encoder. The new semantic embedding α'_0 is obtained by inputting the control language ℓ' corresponding to α'_0 into the BERT. For example, we can use the semantic embedding α'_0 corresponding to the language prior ℓ' 'It is a low-contrast digit-0' to replace the original semantic embedding α_0 corresponding to the language prior ℓ 'It is a high-contrast digit-0'. However, in many practical applications, we have little information about the original target, which means that we can give a complete and accurate language prior ℓ' to get the corresponding true semantic embedding α'_0. For example, we do not know the object is a digit-0, but we want to turn its contrast to low. To solve the above problem, we designed a network named 'Convertor' specifically, which can indirectly obtain the new semantic embedding α'_0 by inputting a more concise control language (alter) with the original semantic embedding α_0. That is to say, the converter provides us with another more general and convenient way to convert the original semantic embedding α_0 into a new semantic embedding α'_0 according to the control language, especially when we know little about the object. It is suitable for situations where we only want to change some of the properties of the reconstruction while keeping others unchanged at the embedding level. For example, the semantic embedding α_0 output by the encoder corresponds to the semantic text ℓ 'It is a low-contrast digit-0', and the control language is 'Keep the shape unchanged, increase the contrast', so the converter integrates these information to output a new semantic embedding α'_0, which corresponds to the new semantic text ℓ' 'It is a high-contrast digit-0'. Obviously, the convertor needs to be trained well in a

supervised way, and the specific loss function for its training is as follows

$$L_{\text{convertor}} = \frac{1}{2}\sum_{i=1}^{M}\left\|C\left(\alpha_{0,i}, \ell_{alter,i}\right) - Bert(\ell_i')\right\|_2^2 \tag{S1.5}$$

where $\ell_{alter,i}$ corresponds to the control language (alter). The above loss function can be directly added to (S1.1) and trained in the first step, as shown in **Supplementary Figure 1.1(a)**. Once the convertor is well-trained, the changed semantic embedding α_0' can be obtained by from the original α_0 and the $\ell_{alter,i}$, i.e., $\alpha_0' = C(\alpha_0, \ell_{alter})$.

Supplementary Figure 1.2(e) is the architecture of the 'Convertor'. The convertor consists of the Long Short-Term Memory (LSTM)[10] and the MLP. Its input is the original semantic embedding α_0 from the encoder and the control language (alter) which is named as ℓ_{alter}, and its output is the new semantic embedding α_0'. The control embedding is extracted from the control language (alter) by the frozen BERT. The feature of the semantic embedding α_0 obtained by MLP is used as the input of LSTM and the control embedding is used as the initial hidden state of LSTM. Then the output of LSTM is processed by MLP to obtain the new semantic embedding α_0'.

In addition, we also show some comparison results in **Supplementary Figure 1.5** before and after fine tuning with the GAN network, which embodies the advantage of the training method of **Supplementary Figure 1.1** in the reconstruction quality and details. The experimental results show that it is necessary to add the 'Discriminator' and finely tune the network using GAN, because it can obviously remove some non-semantic actions and abnormal conditions, such as gallows and blur.

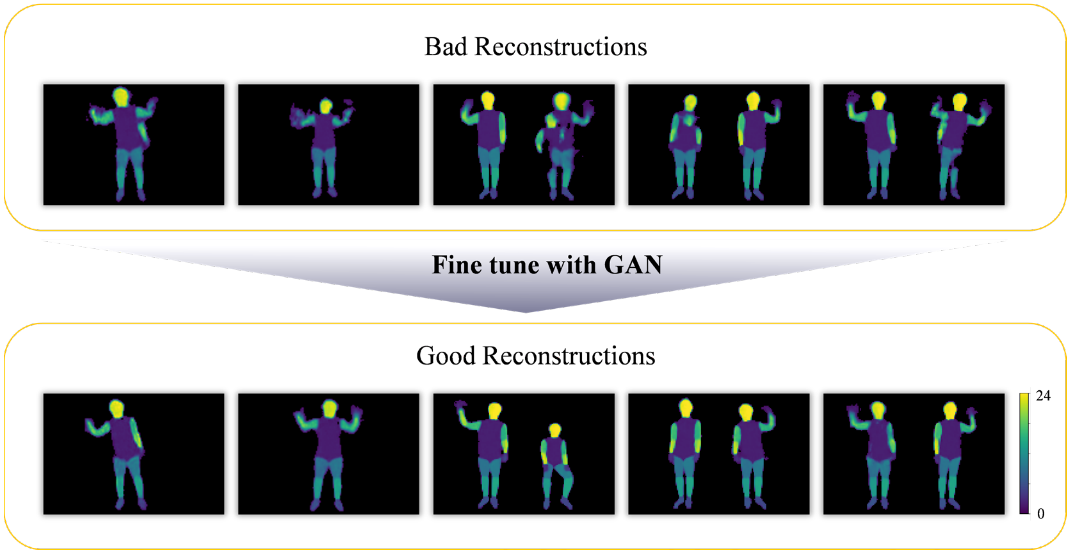

Supplementary Figure 1.5 | A set of comparison results before and after fine-tuning using the GAN network. (Top row) Poor results obtained without fine-tuning using GAN networks. (Bottom row) The corresponding improved result after fine-tuning by the GAN network.

Supplementary Note 2. Comparison of proposed semantic regularization with existing data-driven regularizations

Semantic regularization is also a form of data-driven regularization, but compared to other data-driven regularization techniques, incorporating semantic control offers greater flexibility. Not only can a well-trained network achieve multiple regularization effects, but semantic control also enhances flexibility and improves generalization performance. In the absence of semantics, our method automatically degrades to a standard data-driven regularization approach. In order to clearly show their differences and connections, we supplement a set of comparative experiments, contrasting neural network-based data-driven regularization with our semantic regularization method in terms of noise resistance and generalization ability. The kinds of neural networks used as well as the parameter scale are almost identical for both methods. In the noise experiment, we use the MNIST dataset with the relative permittivity ranging from [2.0, 5.0] for training and testing. In the generalization experiments, we use the MNIST dataset with the relative permittivity ranging from [1.5, 2.0] for training and then directly test with the dataset of geometric shapes with the relative permittivity ranging from [2.0, 3.0], similar to what we did in **Supplementary Note 7**.

(a)

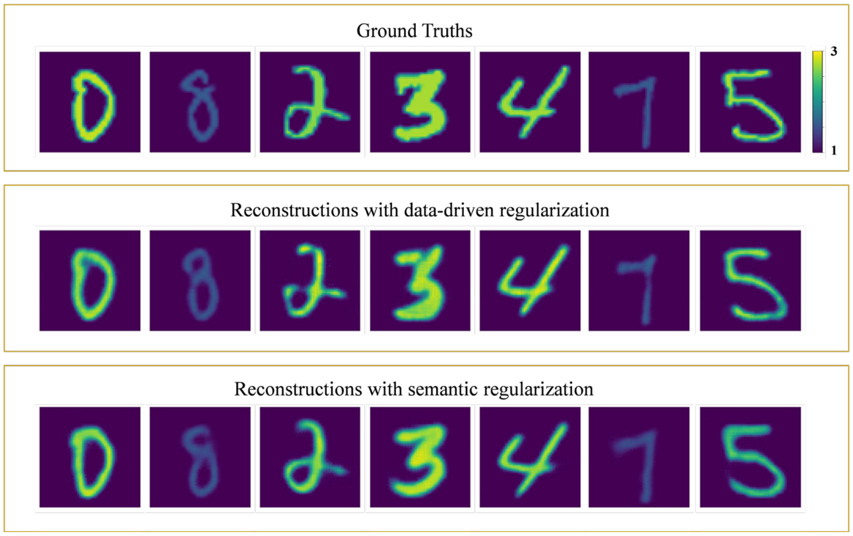

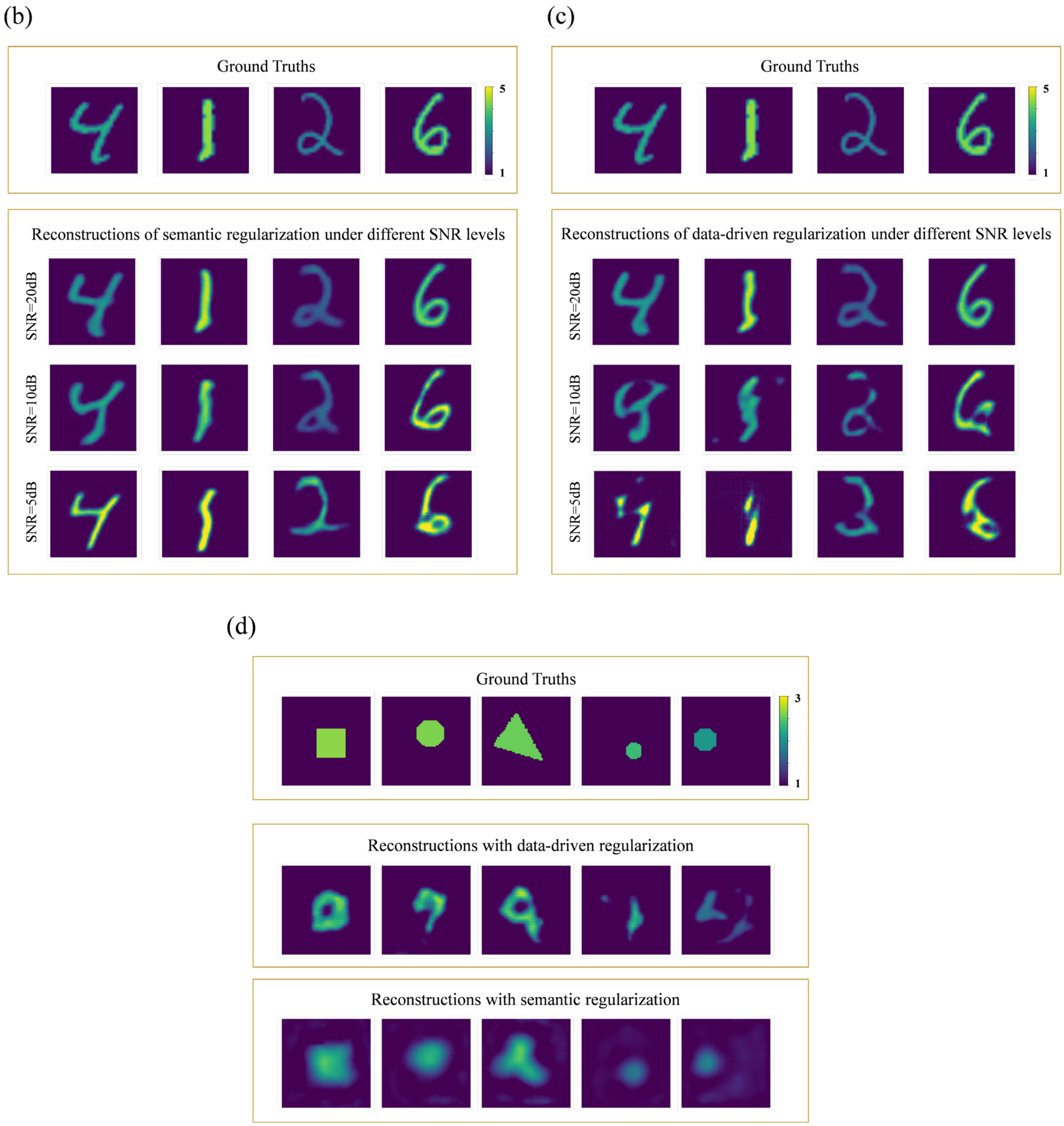

Supplementary Figure 2.1 | Comparison of reconstruction results between common neural-network-based data-driven regularization method and semantic regularization method. (a) Comparison of results for reconstruction accuracy. **(b-c)** Comparison of results for the noise experiment. **(d)** Comparison of results for generalization experiment.

From the results shown in **Supplementary Figure 2.1**, it appears that the semantic regularization and the purely data-driven regularization should exhibit similar effect of reconstruction in the absence of noise. However, under the influence of semantic regularization, semantic imaging

demonstrates greater noise resistance. Additionally, semantic regularization shows stronger generalization capabilities, possibly because semantic alignment operations during training enable the network to better capture semantic-related information while disregarding redundant information in the data. More importantly, we can control the semantics as needed to obtain the reconstruction of the same measurement under different semantics using the same network. However, purely data-driven networks can learn implicit regularization from the data, but we cannot control this process, and the interpretability is poor. Moreover, redundant information in the dataset may also be learned by the network, leading to bad generalization.

Finally, we also add another set of experiments, a contrast with the regularization method based on Principal Component Analysis (PCA) dimensionality reduction, also a classic data-driven regularization approach. Compared with the traditional data-driven regularization based on PCA, one thing of notice is that semantic regularization has significant advantages in reconstruction accuracy, which is largely attributed to the powerful generation ability of the decoder network. Especially when the semantic is changed, the semantic regularization can get better reconstruction effect, while the reconstruction of PCA-based regularization has poor shape under the corresponding basis of other numbers. Some representative results are shown in **Supplementary Figure 2.2**.

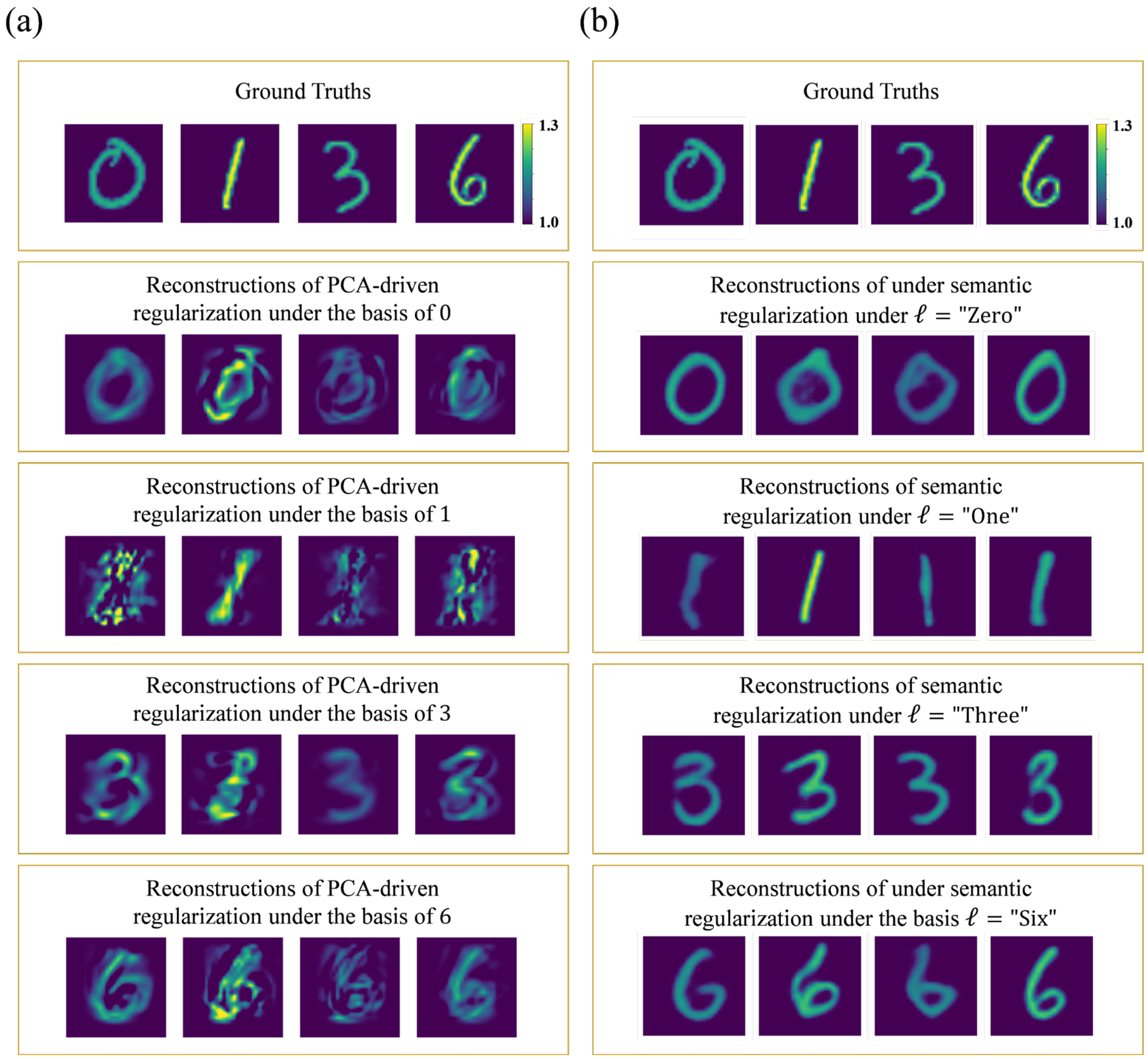

Supplementary Figure 2.2 | Comparison of reconstruction results between common PCA-based data-driven regularization method and semantic regularization method. (a) Reconstruction results of the PCA-based regularization method under bases for different semantics. **(b)** Reconstruction results of the semantic regularization method under different semantics.

Supplementary Note 3. Setups of inverse-scattering experiments

We use the numbers in the MNIST[11] dataset or/and geometric-shape-like to model 2D scatterers as the object in the electromagnetic (EM) inverse scattering problem. The size of the domain of interest (DoI) is 1.28m×1.28m, which is discretized into 64×64 pixels with the size of 0.02m×0.02m. The operating frequency is 300MHz. A total of four transmitters and eight receivers are uniformly placed on a circle with a radius of 2m, as shown in **Supplementary Figure 3.1(a)**. Thus, the received microwave measurement contains 32 complex values. We split each value into real and imaginary parts and reshape it into an 8×8 vector for plotting in this article, such as in **Supplementary Figure 3.1(b)**, where the left half corresponds to the real part (8×4) and the right half corresponds to the imaginary part (8×4). In these cases, the scatterers are assumed to be isotropic and lossless, and the background medium is set as air. The relative permittivity of the scatterer is randomly chosen in the range of [2.0, 5.0] unless otherwise specified. We implement the forward solver based on BCGS-FFT[12] to simulate the measured scattered field data.

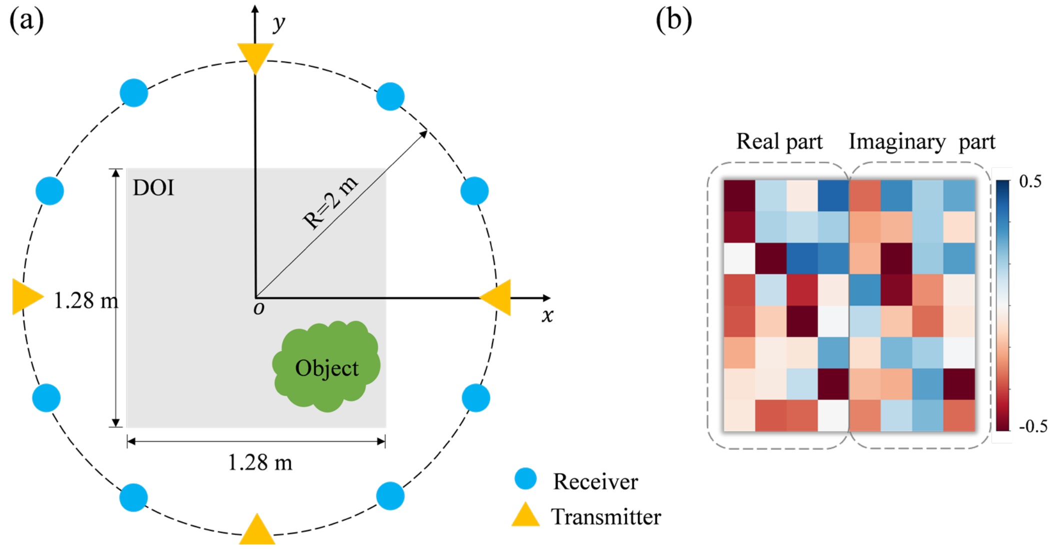

Supplementary Figure 3.1 | Details of numerical experiments for the EM inverse scattering problem. (a) Model configuration for the EM inverse problem scenario. **(b)** Visualization of the microwave measurement y.

Supplementary Note 4. Supplementary inverse-scattering results for Fig. 2e

The language priors including the true semantics of the real objects and the changed semantics for different reconstructions in **Fig. 2e** is illustrated in **Supplementary Figure 4.1(a)**. Here, the language priors of the four real objects in the second row stand of Fig. 4e stand for their four true semantics respectively, and the languages of the reconstruction results in third to sixth rows correspond to these four semantics after artificially changed.

(a)

Index	Language prior
(2,1)	This is a digit-2 like object.
(2,2)	This is a digit-4 like object.
(2,3)	This is a digit-5 like object.
(2,4)	This is a digit-9 like object.
(3,1)	This is a digit-2 like object.
(3,2)	This is a digit-2 like object.
(3,3)	This is a digit-2 like object.
(3,4)	This is a digit-2 like object
(4,1)	This is a digit-4 like object.
(4,2)	This is a digit-4 like object.
(4,3)	This is a digit-4 like object.
(4,4)	This is a digit-4 like object.
(5,1)	This is a digit-5 like object.
(5,2)	This is a digit-5 like object.
(5,3)	This is a digit-5 like object.
(5,4)	This is a digit-5 like object.
(6,1)	This is a digit-9 like object.
(6,2)	This is a digit-9 like object.
(6,3)	This is a digit-9 like object.
(6,4)	This is a digit-9 like object.

(b)

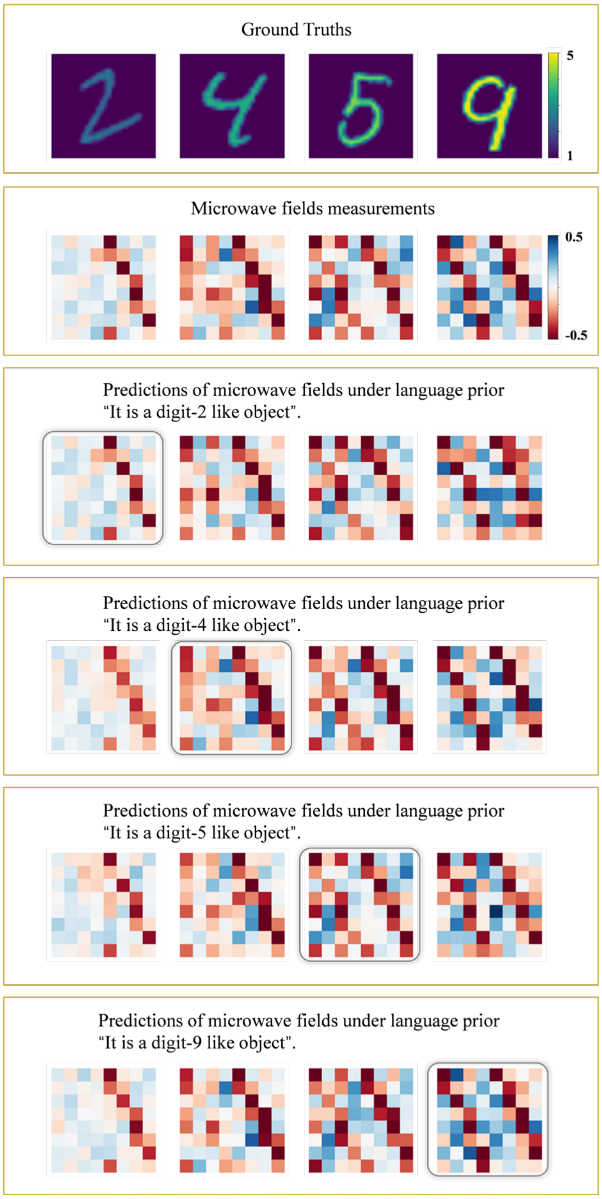

Supplementary Figure 4.1 | Supplementary results of Fig. 2e. (a) Language priors corresponding to different reconstruction results in Fig. 2e. 'Index' represents the corresponding position of Fig. 2e. For example, 'Index (2,1)' indicates the semantic corresponding to the object in the first column and the second row of Fig. 2e. **(b)** The predicted

EM responses corresponding to reconstruction results in Fig. 2e.

For each of the 16 reconstructions in **Fig. 2e**, we calculate the corresponding predicted forward scattered field $\hat{y}$ (i.e., the EM response) using the same model in **Supplementary Figure 3.1**, and show the results in **Supplementary Figure 4.1(b)**. We can find that only when the applied semantic is consistent with the true semantic of the object, the predicted EM response is most similar to the true microwave measurement, thus leading to the smallest data misfit in **Fig. 2d**.

Supplementary Note 5. Noise immunity experiments

Most inverse problems have the property that noise gets amplified during the reconstruction. In this section we test the anti-noise ability of the proposed method. The scattered fields are added with different levels of Gaussian noise for testing. The training data for this experiment is identical to the data used for generating the numerical results presented in **Supplementary Note 3**. In other words, the original microwave measurements used for training are free from any noise contamination. Here, the noise level is defined according to the signal to noise ratio (SNR) of power. In the testing phase, we directly add 30dB, 20dB, 10dB and 5dB (the smaller the value, the stronger the noise) of noise to the microwave measurements. The results of the noise numerical experiment are presented in **Supplementary Figure 5.1** to verify the influence of different levels of noise in the reconstruction process. As shown in Supplementary Figure 5.1, the semantic regularization method achieves a very convincing performances under noisy test conditions: almost no degradation for SNR of 20 dB, small distortions of the results for SNR of 10 dB, and significant distortions for SNR of 5 dB. Nonetheless, even for SNR = 5 dB, the original basic outline and meaning are maintained and recognizable in the output. We hypothesize that the semantic regularization in the training process enables the network to more easily grasp the semantically related information, while ignoring the noisy misleading information in the data, resulting in the impact of noise being reduced. Of course, the reconstruction results corresponding to same microwave data but different semantics also deteriorate with increasing noise levels in similar regularity, as shown in **Supplementary Figure 5.1 (b)**. Moreover, **Supplementary Figure 5.1(c)** to **(e)** show the variation curves of the data misfit for scattered fields, mean square error (MSE) of reconstruction results and accuracy rate of semantic recognition with different noise levels respectively, which are obtained statistically on more than 10^4 test samples. It can be seen that overall with the noise level increasing, the data misfit and MSE gradually rise while the accuracy of semantic recognition gradually decreases at a slow rate. Further, we can observe that these curves change very little at the SNR less than or equal to 20dB, indicating that the proposed network has good performance at SNR level of 20dB. At 10dB SNR, the data misfit and MSE remain at low levels and the predicted semantics remain highly accurate. Even at 5dB noise, the data misfit, MSE, and semantic accuracy are not as bad as expected. The above results indicate that our method has a strong anti-noise ability, which is benefited from the fact that the semantic regularization has a strong capacity to constrain the solution space.

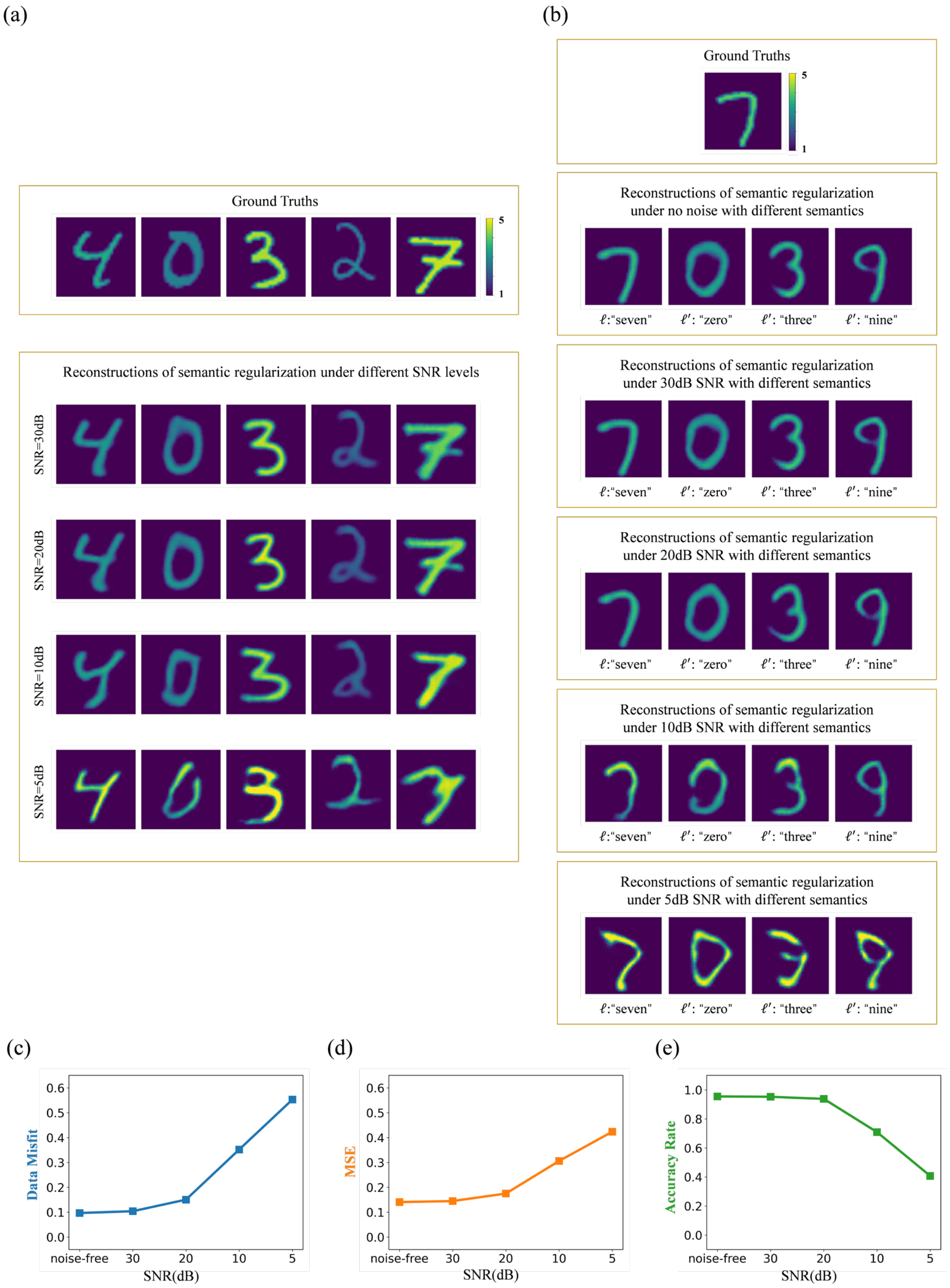

Supplementary Figure 5.1 | Noise experiment results. (a) Reconstruction results for multiple targets under different noises. **(b)** Reconstruction results for different semantics under different levels of noise. **(c)** Data misfit at different levels of noise. **(d)** MSE at different levels of noise. **(e)** Accuracy rate at different levels of noise.

Supplementary Note 6. Comparison of semantic regularization with other regularizations such as L1, L2 and TV

Since we proposed semantic regularization in our method, here we show and compare the results obtained with different regularizations, including L1 norm, L2 norm, Total Variation (TV) norm and semantic regularization, as shown in **Supplementary Figure 6.1**. We used more than 1000 objects from MNIST for testing and randomly plotted some representative results. In the field of EM inverse scattering, the most commonly used regularization is the L2 norm regularization, which expects the target parameter to be as small and smooth as possible, and solving the problem is to optimize the following equation

$$\mathrm{argmin}_x J(x) = [\| y - f(x)\|_2^2 + \gamma \|x\|_2^2], \tag{S6.1}$$

The L2 norm regularization has the advantage of easy solving, but it often underestimates the parameters (e.g. permittivity) of the object and overestimates the shape of the object, so the obtained targets are usually too rounded and blurred, as shown in the third row of Supplementary Figure 6.1. The L1 norm regularization expects the target parameter to be as sparse as possible, and solving the problem is to optimize the following equation

$$\mathrm{argmin}_x J(x) = [\| y - f(x)\|_2^2 + \gamma \|x\|_1^1], \tag{S6.2}$$

The L1 norm regularization is suitable for the reconstruction of sparse targets, but the obtained targets are usually too sparse, which makes the parameters of the targets overestimated, as shown in the row columns of Supplementary Figure 6.1. The TV norm regularization expects smooth and sharply bounded targets, and solving the problem is to optimize the following equation

$$\mathrm{argmin}_x J(x) = \left[\| y - f(x)\|_2^2 + \gamma \left\| \sqrt{(D_x x)^2 + (D_y x)^2} \right\|_1^1\right], \tag{S6.3}$$

where D_x and D_y are the discrete difference matrices in x and y directions, respectively. But the TV norm regularization may obtain too smooth targets, as shown in the fourth row of Supplementary Figure 6.1. The semantic regularization expects to get objects that match the semantics, and solving the problem is to optimize Eq. (1). In general, all three regularization methods sacrifice some reconstruction quality. As is shown in the bottom row of Supplementary Figure 6.1, our semantic regularization can directly lead to the most intuitive solution that conforms to the semantics more intuitively. We can observe that semantic regularization yields better reconstruction results than other regularization. In this experiment, the L1, L2 and TV norm regularization are solved using the Born Iterative Method (BIM).

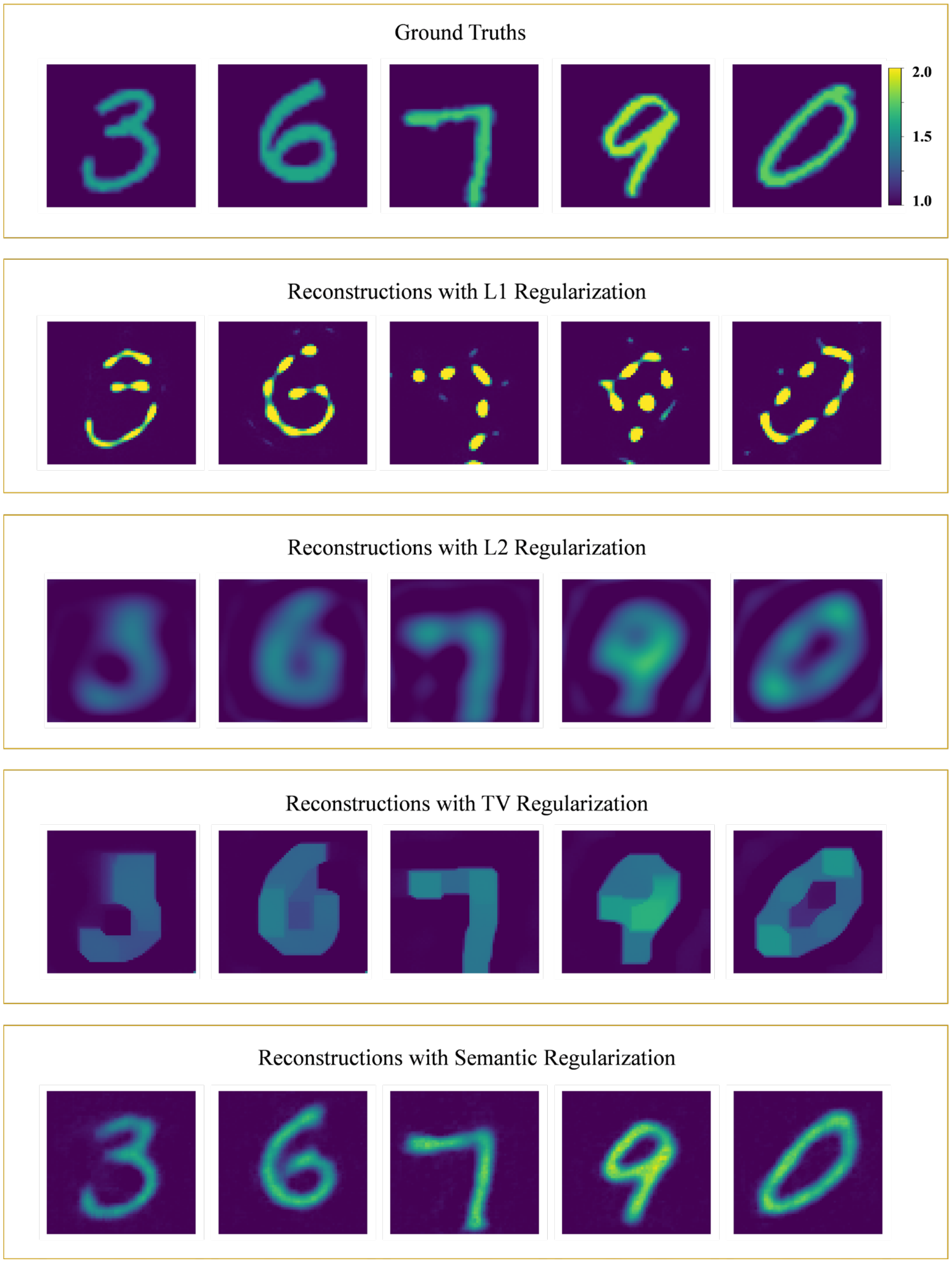

Supplementary Figure 6.1 | Reconstruction results of five selected objects obtained by L1 (second row), L2 (third row), TV (fourth row) and semantic regularization (bottom row) with their ground truths (top row).

Supplementary Note 7. Generalization experiment for EM inverse problem

As we discussed in the article, the proposed network based on semantic regularization can obtain solutions of different meanings under the same measurement by flexibly controlling semantics without changing the network structure. Besides, the addition of semantics also helps to obtain better generalization performance. In this section, a series of numerical experiments is conducted to preliminarily validate the robust generalization of the proposed method which is expected to serve as a general approach for implementing various functions, encompassing L1, L2 and TV regularization effects.

Here, the scattering system remains consistent with what is described in **Supplementary Note 3**, and the training dataset is also comprised of digits in the MNIST dataset. Note that the relative permittivity of the scatterer is randomly chosen in the range of [1.5, 2.0] in the training dataset. We adopt the reconstruction results of the L1 norm, L2 norm and TV norm regularization obtained by BIM shown in the second to fourth rows of **Supplementary Figure 6.1** as the ground truths for network training. Then we use the semantic "The target should be smooth." to represent L2 norm regularization, "The target should be sparse." for the L1 norm regularization, and "The boundary of the target should be sharp." for the TV norm regularization. The semantic embedding α_0 is the embeddings of the [CLS] token of the text output from the BERT with a dimension of 768. Upon completion of training, the encoder-decoder network remains frozen, and we proceed to assess its generalization by employing geometric shapes that are entirely distinct from the MNIST training dataset. That is, we directly use the microwave measurements corresponding to these new objects as input to verify whether it can achieve the same regularization effects as the training dataset under different semantics. These shapes used for testing dataset present significant differences from the training dataset of numbers, and the reconstructions are visualized in **Supplementary Figure 7.1**. Here, **Supplementary Figure 7.1(a)** shows test results where the shape is out of the training data but the relative permittivity falls within the training dataset range ([1.5, 2.0]). **Supplementary Figure 7.1(b)** shows results where the shape is out of the training data and the relative permittivity is slightly out of the training data, with a relative permittivity ranging from [2.0, 3.0]. **Supplementary Figure 7.1(c)** shows reconstructions where the shape is out of the training data and the relative permittivity is far out of the training data, ranging up to [3.0, 5.0]. We can observe the following phenomena:

(i) Semantic regularization can achieve the effects of various existing regularization methods based on their distinctive features. For example, under the semantic of "The target should be smooth", the square is reconstructed to be the circle; under the semantic of "The boundary of the target should be sharp", the circle is reconstructed to be the square; and under "The target should be sparse" semantic corresponding to L1 norm regularization, the object is reconstructed to be some sparse points. (ii) The proposed network based on semantic regularization has a strong generalization ability even when dealing with targets whose shapes and parameters are completely outside the training dataset. In other words, the effect of regularization trained on one type of dataset can even be directly transferred to other datasets even without fine-tuning. These exciting results prove that our semantic regularization method is promising to be a versatile method with strong generalization. It operates independently of dataset content and does not rely heavily on data matching and fitting, a characteristic often associated with traditional deep learning networks and other data-driven regularizations.

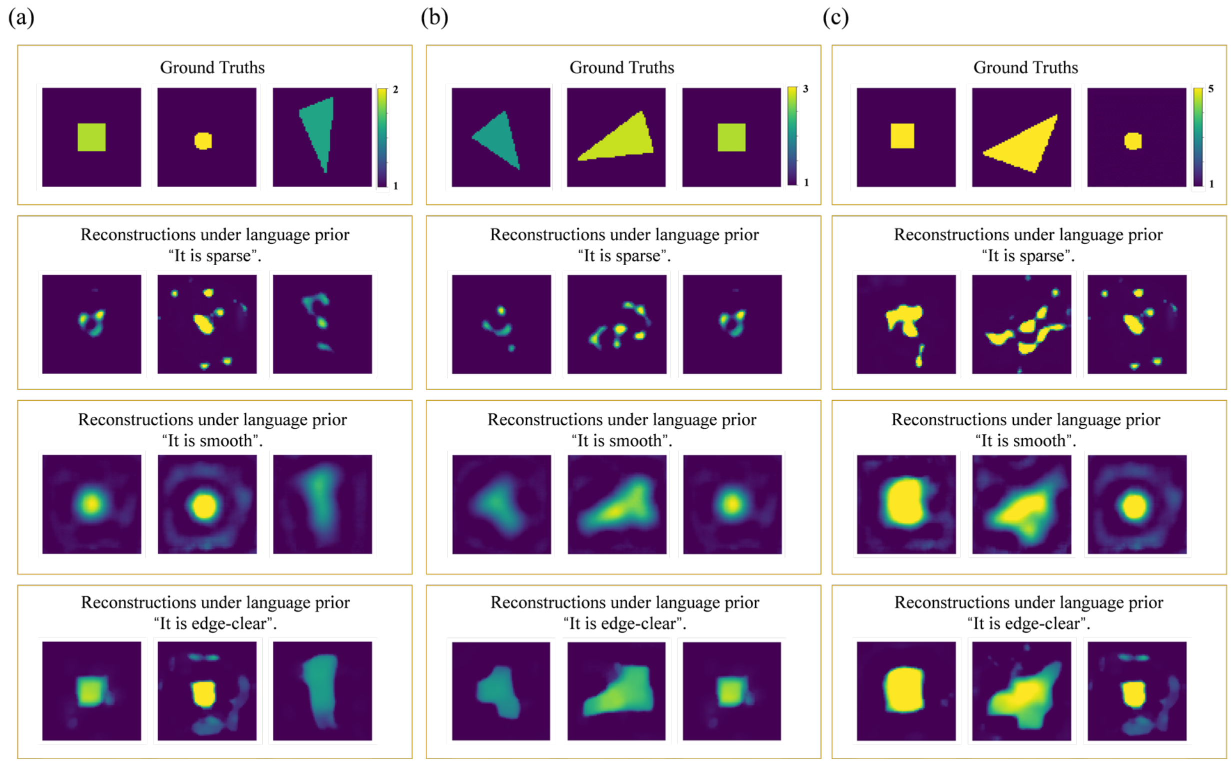

Supplementary Figure 7.1 | Reconstruction results of the geometric targets with different degrees of difference from the training dataset, i.e., (a) Only shapes are outside the training dataset **(b)** Both shape and relative permittivity are outside the training dataset, and the relative permittivity ranges from [2.0, 3.0]. **(c)** Both shape and relative permittivity are outside the training dataset, and the relative permittivity ranges from [3.0, 5.0].

Supplementary Note 8. Evaluation of the semantic regularization with different influence factors

As is well recognized in the literature, differences in hardware and software environments, or changes in experimental settings, may lead to differences in results. So on the one hand, we have provided some code and data as well as a working demo in the supplementary code file for other researchers to refer to. On the one hand, we have conducted a series of controlled experiments to discuss the influence of different factors on the model. All the results below are for the numerical 2D inverse scattering experiment, employing the MNIST dataset with the identical model and setup as described in Supplementary Note 1 and 3.

Table S8.1. MSE on test sets for different optimizers.

Optimizer	SGD	Adam	AdamW	RMSprop	Adagrad
MSE	0.0403	**0.0044**	0.0046	0.0068	0.0079

In the first experiment, except for the optimizer, all other hyperparameters remain the same or are set to default values. From **Table S8.1**, it can be observed that using optimizers from the Adam family yields the lowest test error, followed by momentum-based optimizers, while SGD performs the worst. Therefore, we recommend prioritizing the use of optimizers from the Adam family.

Table S8.2. MSE on test sets for different learning rates.

Learning Rate	1e-2	5e-3	1e-3	5e-4	1e-4
MSE	0.0078	0.0056	**0.0042**	0.0043	0.0048

Table S8.3. MSE on test sets for different batch sizes.

Batch Size	50	100	300	500	1000
MSE	0.0049	0.0049	0.0049	0.0047	**0.0045**

Table S8.4. MSE on test sets for different training epochs.

Epoch	300	500	1000	2000	3000
MSE	0.0047	0.0045	0.0045	**0.0043**	0.0045

Table S8.5. MSE on test sets for different initial values. The five initial values are chosen at random.

Initial Value	Value1	Value2	Value3	Value4	Value5

MSE	**0.0042**	0.0044	0.0044	0.0043	0.0043

Then, we investigated the impact of hyperparameters such as learning rate, batch size, epoch, and initial value while maintaining other hyperparameters constant, using the AdamW optimizer as an example. Results from T**able S8.2 to S8.5** indicate that variations in batch size, learning rate, and initial network weights have minimal influence on the test error, and a low MSE can be obtained as long as the learning rate is not too large. In addition, after the epoch reaches 300, the training epoch has little effect on the model.

Table S8.6. MSE on test sets for different kind of models.

Model	FCs + one-layer ConvT (1.19M)	FCs + two-layer narrow U-net (1.20M)	FCs + four-layer narrow U-net (1.85M)	FCs + four-layer wide U-net (3.31M)	FCs + five-layer wide U-net (26.83M)
MSE	0.0148	0.0056	0.0044	0.0070	0.0075

Finally, we investigated the impact of different network architectures and scales on model accuracy while keeping other hyperparameters constant. As shown in **Table S8.6**, the first model consists of multiple layers of fully connected layers (FCs) and one layer of deconvolutional network (ConvT), with a network parameter count of 1.19M. The second network comprises FCs and two layers of narrower U-net, with a network parameter count of 1.20M. The third network, which is the architecture adopted in this paper, consists of FCs and four layers of narrower U-net, with a network parameter count of 1.85M. The fourth network consists of FCs and four layers of wider U-net, with a network parameter count of 3.31M. The fifth network comprises FCs and five layers of wider U-net, with a network parameter count of 26.83M. From these results, the following observations can be made: i) U-net plays a significant role in improving model performance; even a small U-net can significantly reduce test error. ii) With the increase of the number of network parameters, the test error decreases first and then increases. This is because overfitting occurs when the number of network parameters is too large, resulting in a larger MSE. The supplementary code file contains the code for the specific structures of the above models.

Supplementary Note 9. System configuration of microwave metasurface camera and details of microwave compressive imaging experiments

Introduction of the system for compressive microwave imaging

The proposed intelligent EM metasurface camera is a software-defined system in favor of the high-frame-rate EM sensing. For the purpose of principle illustration, the intelligent EM metasurface camera is designed to work at around commodity Wi-Fi frequency of 2.4GHz, and is used for monitoring human behaviors in indoor environment. With reference to **Supplementary Figure 9.1(a)**, on hardware level the proposed EM metasurface camera consists of a large-aperture programmable metasurface, a field programmable gate array (FPGA), a low-cost commercial software-defined radio device (Ettus USRP X310), a transmitting antenna, a three-antenna receiver and a personal computer. The host computer serves as the brain of the whole system, which is mainly responsible for the instant processing of data stream, calculation of metasurface's control patterns, and communication with other hardware via Ethernet. The USRP along with horn antennas is responsible for generating the probing signals and acquiring the echoes reflected from the target. Both the USRP and metasurface are communicated with the host computer via the Ethernet under the transmission control protocol (TCP); meanwhile, the USRP has I/O series communication with the metasurface. The host computer can calculate the control patterns and send these patterns to the metasurface through FPGA module; at the same time, it sends a command signal to the USRP for synchronizing its transmitting and receiving channel. To trade-off the imaging quality with efficiency, we explore 20 patterns for compressive microwave measurement per frame in this work. For each control pattern, the USRP under control of the host computer will generate the radio signal with chirp waveform, radiate it into the investigation domain through the transmitting antenna, and receive the echoes reflected from the target. It takes 0.01ms to complete this whole measurement procedure; however, it costs 3ms due to the USRP's inherent limitation in our experiments. Thus, it will take 3ms×20=60ms to produce a frame of data, implying the frame rate achievable is about 16 Hz. Afterwards, the acquired echoes are processed by artificial neural networks in the host computer, which is directly responsible for the object reconstruction and recognition. In this work, the chip signal waveform transmitted by the USRP reads:

$$s(t) = \exp(j(2\pi f_c t + \pi K t^2)), \quad 0 \le t \le T \tag{S9.1}$$

where $j = \sqrt{-1}$, $f_c = 2.424\text{GHz}$ is the carrier frequency, $K = B/T$ denotes the sweep rate of

the chirp, B=50MHz is the frequency bandwidth, and T=10μs is the Chirp pulse duration. On software level, the algorithms installed in the host computer are roughly divided into three groups: one is utilized to control the software-defined hardware, like, USRP, FPGA, etc., other is for the signal-level preprocessing and feature extraction, and one is for the machine-learning-driven data postprocessing. The experimental scenario in our lab is shown in **Supplementary Figure 9.1(b)**.

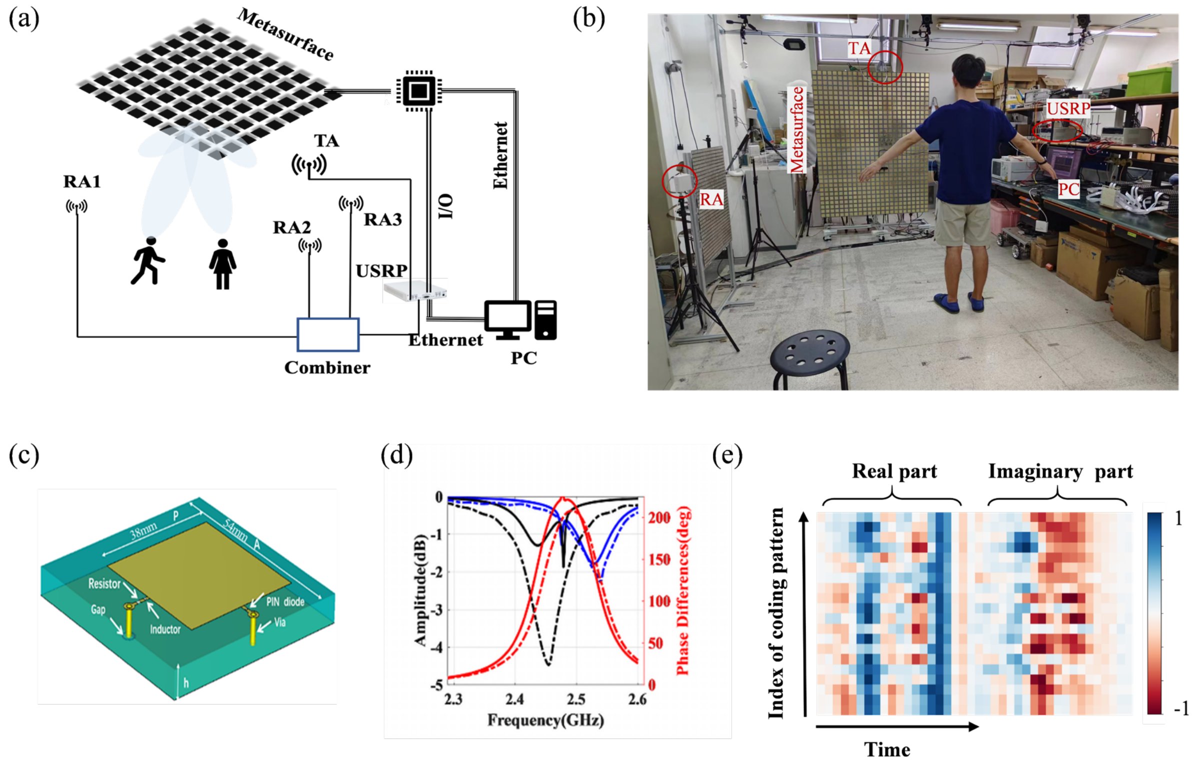

Supplementary Figure 9.1 | System configuration of the proposed intelligent EM metasurface camera working at around 2.4GHz[14,15]. **(a)** The sketch map of proposed intelligent EM metasurface camera system, which is composed of a large-aperture programmable metasurface, a USRP X310, a transmitting antenna (TA), a three-antenna (RA1, RA2, RA3) receiver and a personal computer (PC). **(b)** The physical picture of measurement configuration in our lab during the data acquisition and testing for imaging. **(c)** The sketched map of designed meta-atom. **(d)** Experimental and simulated results of magnitude-frequency and phase-frequency responses of the designed meta-atom. **(e)** Visualization of the microwave measurement after preprocessing.

Next, we would like to elaborate on programmable metasurface, as the core of the proposed intelligent microwave metasurface camera. It is a kind of engineered ultrathin material, which consists of a 2D array of controllable meta-atoms. Here, the programmable metasurface controlled with artificial neural networks is utilized for two major purposes: one is manipulating adaptively the EM wavefields towards the target according to requirements, suppressing the unwanted disturbances from surrounding environment like walls, furnishings, and so on, and the other is

serving as an electronically-controllable coding aperture in compressive-sensing manner. In our implementation, the whole programmable metasurface is designed to be composed of 3×4 identical metasurface panels, and each panel has 8×8 meta-atoms with size of $54\times54mm^2$. Thus, the RM with frequency of 2.4GHz is composed of independently-controllable 32×24 meta-atoms, and its aperture size is $1.7 \times 1.3m^2$. As is shown in **Supplementary Figure 9.1(c),** the 2.4GHz meta-atom has two substrate layers: the top substrate is F4B with a relative permittivity of 2.55 and a loss tangent of 0.0019, and the bottom substrate is FR4. F4B is the abbreviation of high frequency base material made from poly tetra fluoroethylene (PTFE). Flame Retardant 4 (FR4) is a class of printed circuit board base material made from a flame-retardant epoxy resin and glass fabric composite. A SMP1345-079LF PIN diode is integrated into the top square patch and connected to the ground plane via a hole. An RF choke with inductance L = 33nH is used to suppress the AC coupling to ground. We have examined the EM performance of the electronically-controllable meta-atoms numerically and experimentally. In numerical simulations, we use a commercial full-wave EM simulator, CST Microwave Transient Simulation Package 2017. Then the meta-atoms have been designed, fabricated and tested. The 2.4GHz meta-atom is tested using a standard waveguide by VNA. From **Supplementary Figure 9.1(d)** we can observe that the reflection phase of the meta-atom experiences 180° phase difference when the PIN diode is switched from ON (OFF) to OFF (ON) in the selected frequencies. The phase change can be accomplished by switching the external DC voltage applied to the PIN diode from 12V to 0V.

The EM metasurface camera is equipped with a FPGA-based Micro-Control-Unit (MCU) for the control pattern and communication with the host computer. The FPGA chip is used to distribute all commands to 768 PIN diodes. The MCU is responsible for dispatching all commands sent from a master computer subject to one common clock (CLK) signal. In our work, the adopted CLK of MCU is 50MHz, and the switching time of PIN diode is about 10us each cycle. Each metasurface panel is equipped with 8 8-bit shift registers (SN74LV595APW), and every 8 PIN diodes are sequentially controlled by the same shift register. Then MCU performs almost real-time manipulations of all PIN diodes by sending the commands over 24 independent branch channels. In addition, 768 red-color LEDs are soldiered to indicate the status of the associated PIN diodes, in particular, to indicate clearly whether the PIN diode works well or not. Each meta-atom soldiered with one PIN diode has binary distinct EM response states. Specifically, the reflection phase

response changes by 180° around 2.4GHz when the PIN diode is switched from OFF (ON) to ON (OFF), while the amplitude remains almost unaltered. The status of PIN diodes is controlled by a FPGA-based micro-control-unit with clock of 50MHz. The power required for programming the metasurface is exceedingly low, often on the order of just a few micro-Watts per meta-atom. Owing to the large-view field nature enabled by the large-aperture programmable metasurface, the target's information can be readily captured by the fixed receivers. Therefore, we expect that the reconstruction of target's information can be readily achieved from the compressive measurements.

Dataset collection and preparation

The compressive microwave imaging experiment is deployed in a real-world indoor environment, resulting in seriously noisy microwave measurements, especially at around 2.4GHz. Therefore, in order to obtain satisfactory imaging results, we perform denoising as well as mean-value filter with respect to the slow time on the original microwave signal received by the receiving antennas, and finally get the microwave measurement y of size 20×40 as the input of the encoder network in **Supplementary Figure 1.2**. As shown in **Supplementary Figure 9.1(e)**, 20 stands for the number of control patterns which is randomly generated in our experiment, and we select the 40 most obvious feature points under each coding pattern, which is formed by concatenating 20 real parts and 20 imaginary parts.

One critical issue to our developed encoder-decoder network is to train it in a supervised way. In order to collect and process the optical labels corresponding to microwave measurements above, a commercial binocular optical camera named ZED2[13] has been synchronized into the EM metasurface camera system. It is also integrated into our EM metasurface camera to acquire the corresponding optical image synchronously with the microwave. However, the original optical image captured by ZED2 contains considerable background information unrelated to the target, necessitating to go through a series of preprocessing before being utilized as optical labels (i.e., ground truths) for compressive microwave imaging. In our experiment, we directly adopt the DensePose-RCNN[16] model to convert the raw optical image taken by the ZED2 into the UV-transformed single-channel image which divides the human body into 24 common parts after background removal, thus obtaining the 'semantically segmented' images as the supervision for network training. As is shown in **Supplementary Figure 9.2(a)**, we use the 24 integer values from '1' to '24' in the color bar to represent 24 parts of the human body such as head, left arm, left thigh,

etc., and the value ‘0’ is used to represent the environmental background. It should be noted that we plot all imaging results using the color bar named ‘viridis’, but we replace its original color when the value is ‘0’ with color ‘black’ in order to clearly distinguish people from the background.

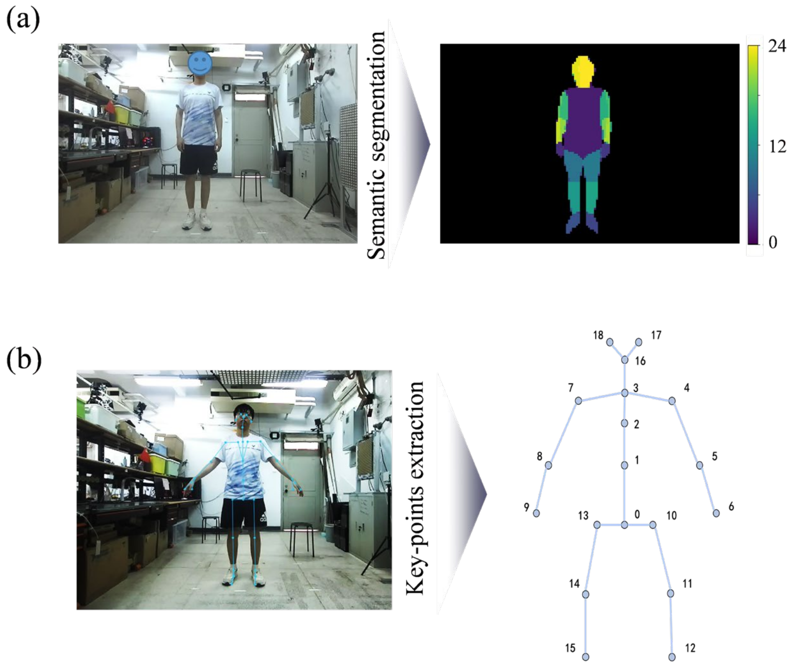

Supplementary Figure 9.2 | The data-processing process of optical data as labels. (a) Semantic segmentation process from optical photo to UV-image. **(b)** The extraction process of 3D skeleton key-points, where 34 main skeleton points are automatically extracted from the photos taken by ZED2 after being processed by its built-in SDK, and the most critical 19 skeleton points are selected as the final 3D skeleton key-point labels.

In our experiment, we collect more than 50000 sets of data which are semantically annotated manually for 2D compressive microwave imaging in both single and multi-person scenarios, 90% of which are for training and 10% for testing. During the semantic annotation phase, we employ terms like ‘left/right’ and ‘far/near’ to describe the subject’s position in the lab, and incorporate common indoor actions like standing, sitting, bending, waving, swinging arms, etc., integrating them with the identity of the subject into complete sentences which can completely describe the behavior. For example, "Sam is swaying their right hand on the left, while Jack is stretching on the right." In cases involving multiple-person imaging, we also collect interactions between different subjects, such as handshakes, hugs and so on.

Supplementary Note 10. Details of 4D compressive microwave meta-imaging experiments

3D visual-semantic map

Here, we elaborate on the 3D visual-semantic map specifically designed for the ill-posed 4D compressive microwave meta-imaging problem mentioned in this paper. In order to construct such a 3D map based on the laboratory indoor environment, we first scan the lab with the ZED2 camera and convert it into the form of 3D point cloud or 3D mesh using the SDK built into ZED2. In cooperation with our LLM-based method, we perform semantic processing on the 3D data. Specifically, we manually annotate various regions with semantic labels, such as 'computer', 'chair', 'cabinet', and we also partition and label different ground areas, thereby constructing a comprehensive semantic map similar to **Fig. 4a**. As a result, given any coordinate, we can directly translate into the corresponding semantic location on the semantic map rather than just use the numerical value of the coordinate that ZED2 can provide. For instance, the coordinate (1.1m, 2.1m, 1.5m) means 'in front of the desk', the coordinate (1.3m, 1.2m, 0.8m) means 'at the left side of the chair'. Therefore, while making and labeling datasets, we can make a more intuitive and specific description of the human behavior based on our semantic map. This approach to modeling semantic maps enables our network to gain a deeper understanding of the experimental scene, facilitating easier integration of semantic regularization to leverage the benefits of language.

Dataset collection and preparation

In the 4D microwave imaging experiments, we directly use the SDK built into ZED2 which enables to accurately extract the 34-key-point 3D skeleton of the subject from the original optical photo. Considering both the imaging speed and accuracy, we ultimately opt for 19 critical points from them. These points encompass the head, trunk, limbs, and other pivotal body regions, as illustrated in **Supplementary Figure 9.2(b)**. Then we combine a sequence of continuous-time skeletons into a video and annotate them with corresponding semantics, resulting in the 4D dataset. In the experiment, we treat every 25 frames as a complete 4D sequence. Subsequently, the original skeletons are filtered and smoothed in time domain to reduce the jitter and outliers of the training dataset. The above methods also apply to the processing data in multiple people scenarios. For example, in our experiments, we use a total of 19×2 points to represent the 3D skeletons of two people, respectively. In addition, the microwave measurements share the same structure and

meaning as depicted in **Supplementary Figure 9.1(e)**. We also combine consecutive microwave measurements into a sequence (also 25 frames like the skeleton), which serves as the input of the encoder network in **Supplementary Figure 1.4**.

Taking into account multi-person imaging in the form of video contains more information and therefore can describe more complex and meaningful actions, in the 4D microwave imaging experiment, we specially select actions with significant body language implications in addition to existing actions mentioned in the dataset of **Supplementary Note 9**. The body languages contained in these movements are widely used in military, rescue, transportation and other fields. For example, 'raise hands and look up' corresponds to the 'SOS' signal, 'put the right hand up and forward' also means 'go forward'. Finally, we collect more than 200000 sets of data for 4D compressive microwave imaging, 90% of which for training and 10% for testing.

Supplementary Note 11. Supplementary results for 4D compressive microwave meta-imaging experiments

Supplementary Figure 11.1(a) provide some selected experimental results of 4D compressive microwave imaging in a single-person scenario, showing wonderful reconstruction and recognition effect and strong privacy protection ability. The classification matrix of 8 different semantics in the second and third row of **Fig. 4b** is shown in **Supplementary Figure 11.1(b)**, in which we used 10,000 samples for testing and statistics. Here, semantic recognition means that we obtain the corresponding semantic embedding through the encoder model according to the input microwave measurement, and then recognize the corresponding semantics of the action after classification. The recognition accuracy of all semantics is above 95%, indicating that the proposed method can produce simultaneously associated high-level semantic recognition results.

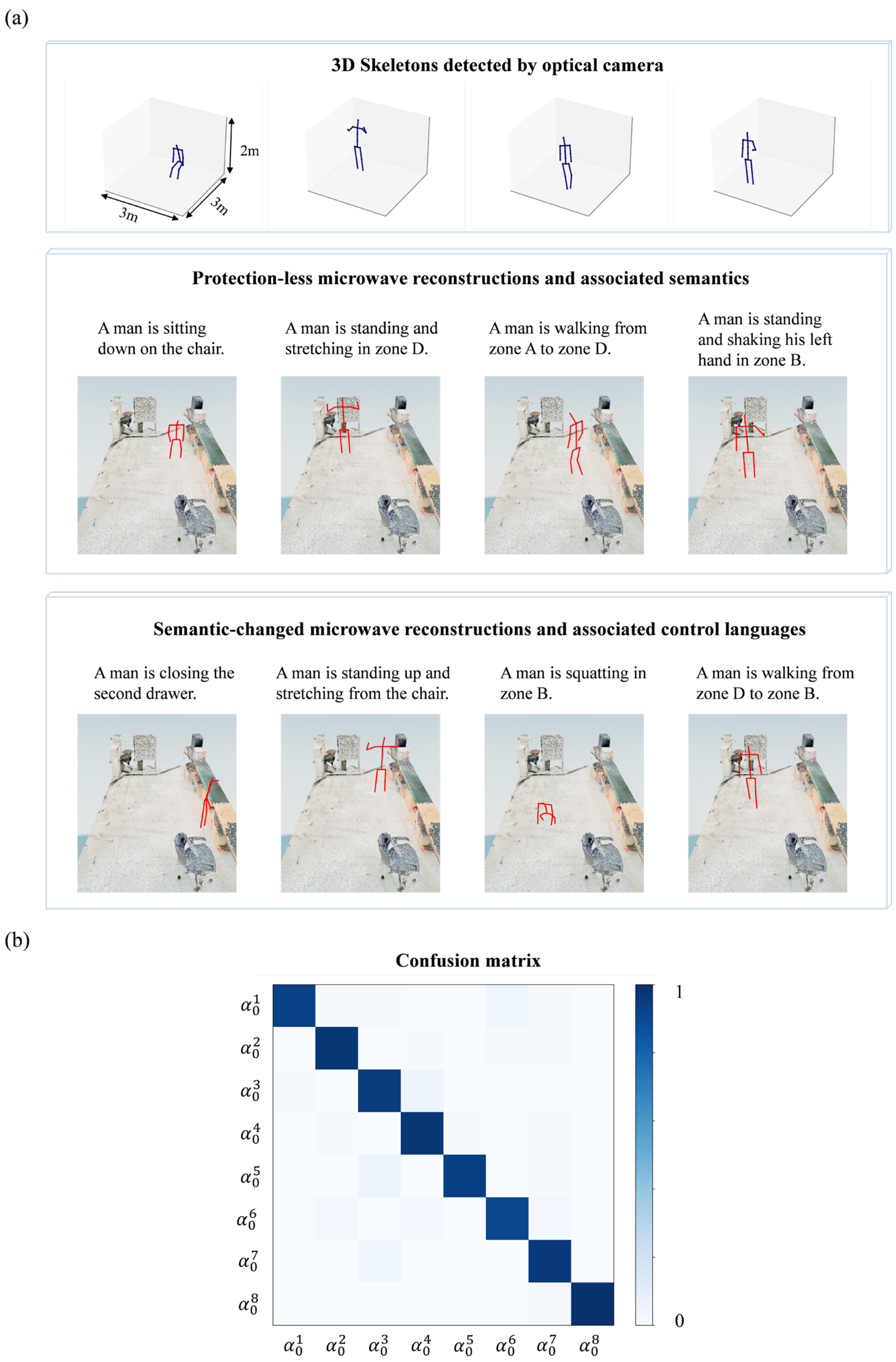

Supplementary Figure 11.1 | Supplementary Results for the 4D Microwave Imaging Experiment. (a) Imaging results under different semantics and microwaves in the case one subject move freely in our lab. **(b)** Confusion matrix for semantic recognition.

Supplementary Note 12. Integration of semantic regularization with iterative inverse-scattering approaches

Semantic regularization can be integrated not only with neural networks, but also with iterative inverse scattering approaches. In this supplementary note, we integrate semantic regularization with the Born Iterative Method (BIM), an iterative inverse scattering method commonly used in the field of EM inverse scattering.

BIM approximately solves a nonlinear EM inverse scattering problem by iteratively alternately solving two linear problems. Formally, the BIM consists of iteratively solving the following linear inverse problem, i.e.,

$$E_s = j\omega\varepsilon_0 G\chi E_t \tag{S12.1}$$

Herein,

$$E_t = E_{in} + j\omega\varepsilon_0 G\chi E_t. \tag{S12.2}$$

Note that Eqs. S12.1 and S12.2 come from Eqs. 4 and 3 in the main text, respectively.

Then, substituting the pre-trained semantic decoder $\chi = D(\Delta\alpha, \alpha_0)$ (α_0 is the prior semantic embedding) into Eq. S12.1, we can arrive at:

$$E_s = j\omega\varepsilon_0 G D(\Delta\alpha, \alpha_0) E_t \tag{S12.3}$$

Here we directly utilize the same network as the 'Decoder' in **Fig. 1** as the semantic decoder, and once its pre-training is completed, we freeze it when solving with BIM. It is clear that Eqs. S12.2 and S12.3 form the foundation of semantic-integrated BIM, as summarized in **Table S12.1**.

Table S12.1. Algorithm of semantic-integrated BIM.

```
Initializing Δα = 0;
While (Not arriving at some stopping criterion)
   DO
      Updating Δα by solving E_s = jωε_0 G D(Δα, α_0) E_t;
      Calculating χ = D(Δα, α_0)
      Updating E_t by solving E_t = E_in + jωε_0 G χ E_t
   END
```

To examine the performance of semantic-integrated BIM, a set of numerical experiments have been conducted and corresponding results have been plotted in **Supplementary Figure 12.1**. It can be immediately observed from **Supplementary Figure 12.1** that the semantic-integrated BIM is

better than standard BIM in terms of the convergence effect and the reconstruction quality.

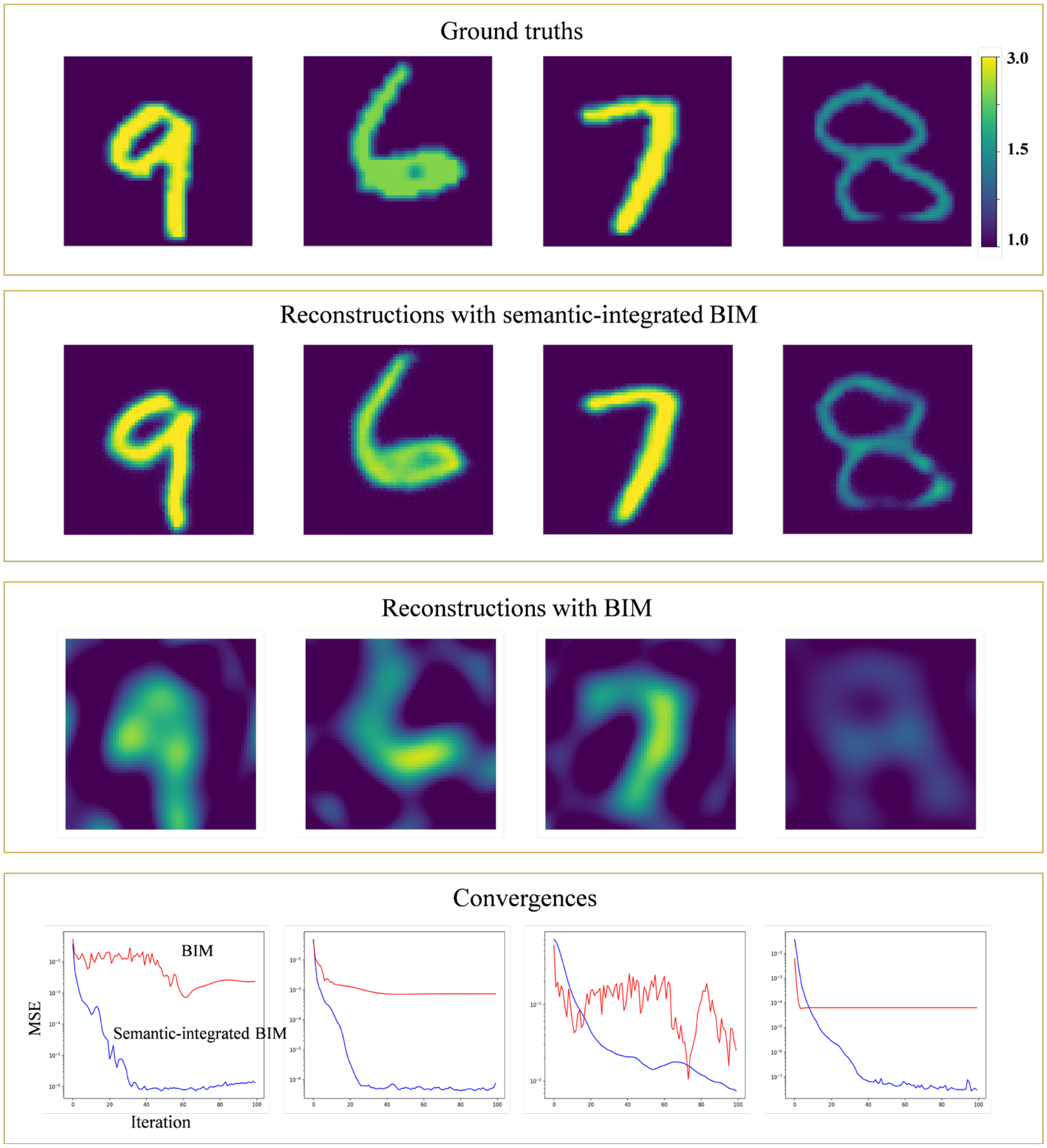

Supplementary Figure 12.1 | Comparison between semantic-integrated BIM and conventional BIM. (top row) ground truths, (second row) reconstructions with semantic-integrated BIM, (third row) reconstructions with conventional BIM, (bottom row) convergence behaviors in terms of MSE vs. iterations.

For the purpose of secure reconstruction, as detailed in main text, we would like to examine the reconstruction quality of semantic-integrated BIM with different control semantics (i.e., α_0), and report a set of numerical results in **Supplementary Figure 12.2**. It can be seen that the semantic-integrated BIM can produce the reconstructions that are matched with specified control semantics,

while the predicted measurements are well consistent with ground truths.

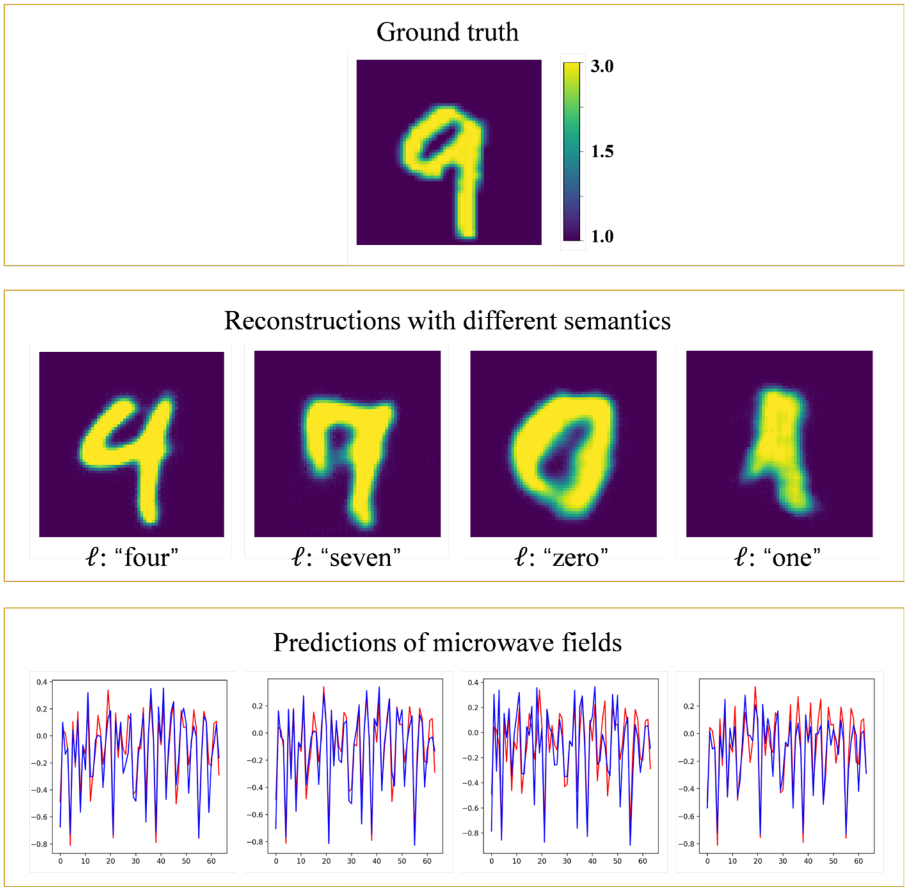

Supplementary Figure 12.2 | Reconstructions of semantic-integrated BIM with different control semantics. (top) ground truth, (middle) reconstructions under different control semantics, (bottom) predictions (red lines) and ground truths (blue lines) of scattering fields corresponding to reconstructions in middle row.

Supplementary Note 13. MSE statistics of the obtained solutions

The MSE on the obtained solutions in all cases when the calculation is possible and meaningful is presented in tabular form as follows, and the 'Row… Column…' in the table indicates the corresponding position of the solution in the figure.

Table S13.1. The MSE of the results under different semantics in Fig. 2c.

ℓ_1	ℓ_2	ℓ_3	ℓ_4	ℓ_5	ℓ_6	ℓ_7
1.116	0.946	0.861	0.554	0.390	0.112	0.101

Table S13.2. The MSE of the results under different objects and semantics in Fig. 2e.

	Column 1	Column 2	Column 3	Column 4
Row 3	0.022	0.359	0.509	0.803
Row 4	0.173	0.050	0.426	0.224
Row 5	0.152	0.341	0.054	0.602
Row 6	0.181	0.166	0.401	0.079

Table S13.3. The MSE of the results under different objects and semantics in Fig. 2g.

	Column 1	Column 2	Column 3	Column 4
Row 2	0.161	0.077	0.237	0.152

Table S13.4. The MSE of the results in Fig. 3b.

	Column 1	Column 2	Column 3	Column 4	Column 5
Row 2	0.004	0.005	0.002	0.006	0.009

Table S13.5. The RMSE (m) of the results in Fig. 4b.

	Column 1	Column 2	Column 3	Column 4
Row 2	0.056	0.038	0.047	0.057

Table S13.6. The MSE of the results under different regularizations and objects in Supplementary Figure 2.1(a).

	Column 1	Column 2	Column 3	Column 4	Column 5	Column 6	Column 7
Row 2	0.078	0.011	0.186	0.287	0.062	0.007	0.131

Row 3	0.112	0.019	0.172	0.165	0.074	0.006	0.131

Table S13.7. The MSE of the results under different noise and objects for semantic regularization in Supplementary Figure 2.1(b).

	Row 2	Row 3	Row 4
Column 1	0.075	0.141	0.236
Column 2	0.059	0.197	0.343
Column 3	0.052	0.074	0.184
Column 4	0.048	0.264	0.657

Table S13.8. The MSE of the results under different noise and objects for data-driven regularization in Supplementary Figure 2.1(c).

	Row 2	Row 3	Row 4
Column 1	0.079	0.235	0.398
Column 2	0.077	0.204	0.440
Column 3	0.039	0.161	0.182
Column 4	0.056	0.521	0.812

Table S13.9. The MSE of the results under different regularizations and objects in Supplementary Figure 2.1(d).

	Column 1	Column 2	Column 3	Column 4	Column 5
Row 2	0.085	0.067	0.050	0.024	0.035
Row 3	0.095	0.058	0.058	0.021	0.021

Table S13.10. The data misfits of the results under different objects and semantics in Supplementary Figure 4.1(b).

	Column 1	Column 2	Column 3	Column 4
Row 3	0.103	0.493	0.382	0.453
Row 4	0.538	0.108	0.415	0.275
Row 5	0.439	0.387	0.062	0.485
Row 6	0.437	0.293	0.355	0.120

Table S13.11. The MSE of the results under different objects and noise in Supplementary Figure 5.1(a).

	Row 2	Row 3	Row 4	Row 5
Column 1	0.050	0.075	0.141	0.236
Column 2	0.034	0.049	0.155	0.354
Column 3	0.098	0.113	0.488	0.712
Column 4	0.057	0.052	0.075	0.285
Column 5	0.302	0.400	0.695	1.309

Table S13.12. The MSE of the results under different noise and semantics in Supplementary Figure 5.1(b).

	Column 1	Column 2	Column 3	Column 4
Row 2	0.024	0.633	0.448	0.189
Row 3	0.025	0.622	0.458	0.194
Row 4	0.052	0.701	0.492	0.270
Row 5	0.191	0.716	0.591	0.365
Row 6	0.703	0.982	0.855	0.688

Table S13.13. The MSE of the results under different objects and regularizations in Supplementary Figure 6.1.

	Column 1	Column 2	Column 3	Column 4	Column 5
Row 2	0.026	0.100	0.090	0.074	0.060
Row 3	0.008	0.013	0.026	0.019	0.018
Row 4	0.009	0.012	0.030	0.021	0.018
Row 5	0.003	0.003	0.009	0.003	0.003

Table S13.14. The RMSE (m) of the results in Supplementary Figure 11.1(a).

	Column 1	Column 2	Column 3	Column 4
Row 2	0.041	0.035	0.045	0.036

Table S13.15. The MSE of the reconstruction results in Supplementary Figure 12.1.

	Column 1	Column 2	Column 3	Column 4
Row 2	0.018	0.008	0.007	0.019
Row 3	0.181	0.137	0.123	0.055

Table S13.16. The data misfits of the results under different semantics in Supplementary Figure 12.2.

	Column 1	Column 2	Column 3	Column 4
Row 3	0.383	0.324	0.419	0.370

Supplementary Note 14. Applying the proposed semantic regularization in other domains (reservoir fluid)

The proposed semantic regularization method can be easily extended to various fields outside electromagnetics, for instance, fluid dynamics. Moreover, the semantic regularization method can also be combined with various traditional physical approaches, as demonstrated in the revised **Supplementary Note 12**. Therefore, we consider the proposed method as a general methodology that can fuse various models and generalize to various domains.

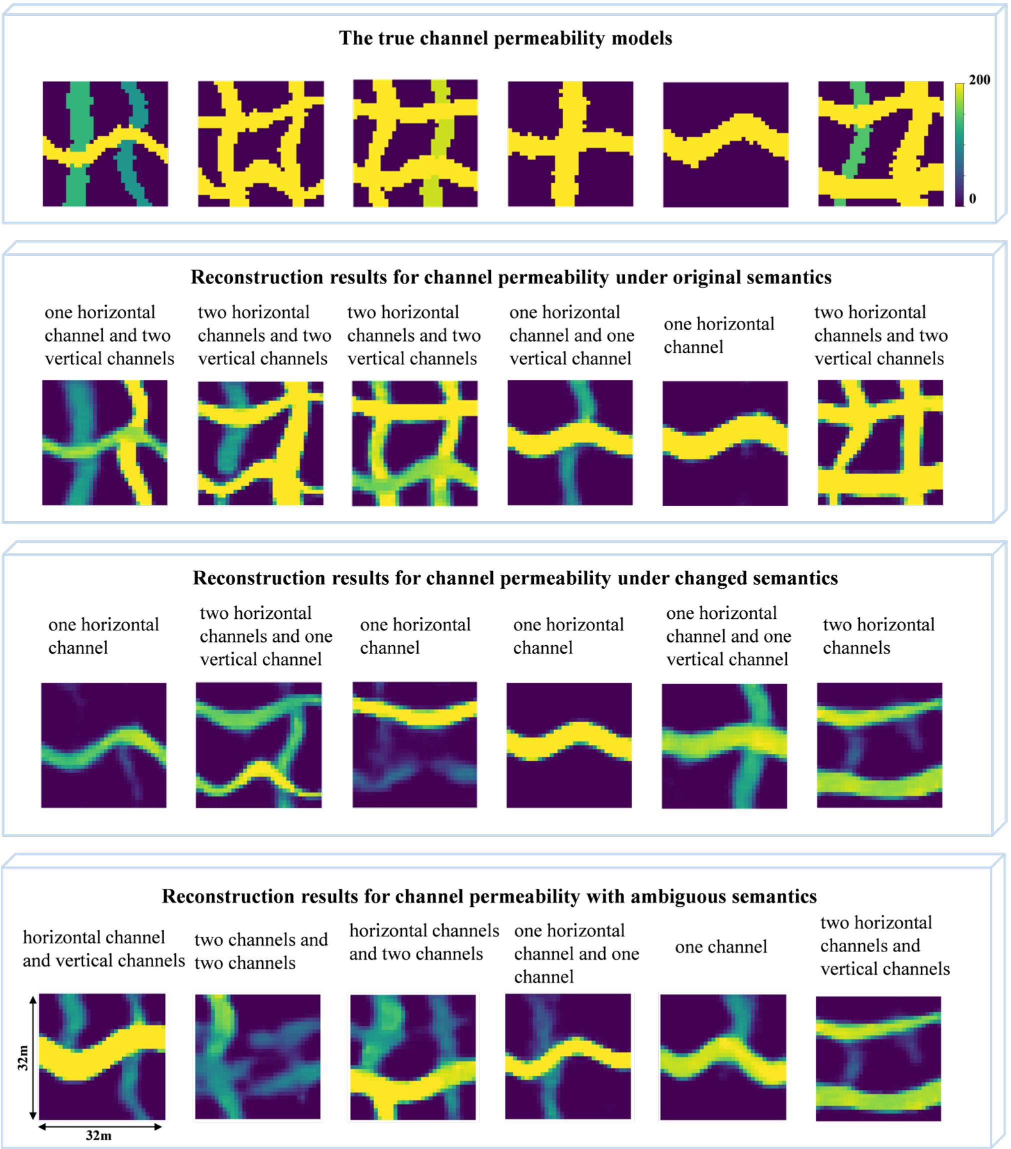

Supplementary Figure 14.1 | Reconstruction results for channel permeability under different observation and semantics.

As an example, above we show the outcomes of a numerical experiment using semantic regularization in the domain of fluid mechanics, i.e., nonlinear subsurface flow inverse problems. It reconstructs spatially variable hydraulic rock properties such as permeability and porosity from dynamic flow field data, thereby helping to accurately predict fluid flow displacement in subsurface environments, which has a wide range of applications such as reservoir simulation. Here, using the forward mathematical method of fluid dynamics in supplementary reference [17,18] to carry out simulation experiments, we generated more than 100,000 sets of channel permeability models with different semantic representations and corresponding measurements of pressure and saturation varying over time. The specific simulation parameters are shown in **Table S14.1**. In the network training phase, we adopt the similar architecture to **Fig. 1** to solve the corresponding inverse problem, that is, to predict the distribution of permeability in this subsurface environment from the observed fluid pressure and saturation in the injection and production wells. This is also an ill-conditioned inverse problem with multiple solutions.

Table S14.1. General simulation information.

Simulation parameters	
Phases	Two-phase (o/w)
Simulation time	1 year
Grid systems	32×32×1
Cell dimensions	1m×1m×1m
Rock porosity	0.2
Initial oil saturation	1
Injection volume	1 PV
Number of injectors	3
Number of producers	4
Assimilation information	
Observation intervals	30 days
Observation at injection wells	Pressure
Observation at production wells	Pressure and saturation

Supplementary Figure 14.1 shows some representative results, from which it can be seen that the permeability distribution can be accurately derived from the observed time series of pressure and saturation. Furthermore, the distribution under different semantics can also be effectively obtained. When altering the number and/or direction of channels through semantics, the model can output the corresponding changed distribution that aligns with the semantic. Additionally, by comparing the results in the second and fourth rows of Supplementary Figure 14.1, it becomes evident that more precise semantics lead to more accurate permeability distributions. For instance, transitioning from the semantics of 'one channel' to 'one horizontal channel' results in the disappearance of the vertical channel, making it more consistent with the ground truth. Therefore, it strongly proves that the proposed semantic regularization method can be applied to various physical fields, promising to become a general method.

Supplementary Video 1. Language-controllable reconstruction of 4D compressive microwave imaging for a single subject.

Supplementary Video 2. Language-controllable reconstruction of 4D compressive microwave imaging for two subjects.

References

[1] Glorot, X., Bordes, A., & Bengio, Y. Deep sparse rectifier neural networks. *Proceedings of the fourteenth international conference on artificial intelligence and statistics* (JMLR Workshop and Conference Proceedings, 2011).

[2] Vaswani, A. et al. Attention is all you need. *Adv. Neural Inf. Process. Syst.* **30** (2017).

[3] http://www.huggingface.co.

[4] Krizhevsky, A., Sutskever, I., & Hinton, G. E. Imagenet classification with deep convolutional neural networks. *Advances in neural information processing systems* (2012).

[5] Ronneberger, O., Fischer, P., & Brox, T. U-net: Convolutional networks for biomedical image segmentation. *Medical Image Computing and Computer-Assisted Intervention–MICCAI 2015: 18th International Conference* (2015).

[6] He, K., Zhang, X., Ren, S., & Sun, J. Deep residual learning for image recognition. *Proceedings of the IEEE conference on computer vision and pattern recognition* (2016).

[7] Rosenblatt, F. The perceptron: a probabilistic model for information storage and organization in the brain.

Psychol. Rev. **65**, 386 (1958)

[8] Tolstikhin, I. O. et al. Mlp-mixer: An all-mlp architecture for vision. *Advances in neural information processing systems* (2021).

[9] Loshchilov, I., & Hutter, F. Decoupled weight decay regularization. *doi.org/10.48550/arXiv.1711.05101* (2017).

[10] Hochreiter, S., & Schmidhuber, J. Long short-term memory. *Neural computation* ***9***, 1735-1780 (1997).

[11] LeCun, Y., Bottou, L., Bengio, Y., & Haffner, P. Gradient-based learning applied to document recognition. *Proceedings of the IEEE* **86**, 2278-2324 (1998).

[12] Lan, T., Liu, N., Liu, Y., Han, F., & Liu, Q. H. 2-D electromagnetic scattering and inverse scattering from magnetodielectric objects based on integral equation method. *IEEE Trans. Antennas Propag.* **67**, 1346-1351 (2018).

[13] https://www.stereolabs.com/zed-2/

[14] Wang, Z., Zhang, H., Zhao, H., Cui, T. J., & Li, L. Intelligent electromagnetic metasurface camera: system design and experimental results. *Nanophotonics* **11**, 2011-2024 (2022).

[15] Li, L., Shuang, Y., Ma, Q. et al. Intelligent metasurface imager and recognizer. *Light Sci Appl* **8**, 97 (2019).

[16] Güler, R. A., Neverova, N., & Kokkinos, I. Densepose: Dense human pose estimation in the wild. *Proceedings of the IEEE conference on computer vision and pattern recognition* (2018).

[17] Aarnes, J. E., Gimse, T., & Lie, K. A. An introduction to the numerics of flow in porous media using Matlab. *Geometric modelling, numerical simulation, and optimization: applied mathematics at SINTEF.* 265-306 (Springer Berlin Heidelberg, 2007).

[18] Li L. & Jafarpour B., Effective solution of nonlinear subsurface flow inverse problems in sparse bases, *Inverse Problems*, 26, 105016 (2010)
