# Supplementary material for: Semantic regularization of electromagnetic inverse problems
Source: Nat Commun. 2024 May 8;15:3869. doi: 10.1038/s41467-024-48115-5 (PMC11079068; doi:10.1038/s41467-024-48115-5)
Supplement: Supplementary file 3 — Description of Additional Supplementary Files [file 41467_2024_48115_MOESM3_ESM.pdf]

Description of Additional Supplementary Files

File Name: Supplementary Video 1

Description: Language-controllable reconstruction of 4D compressive microwave imaging for a single subject.

File Name: Supplementary Video 2

Description: Language-controllable reconstruction of 4D compressive microwave imaging for two subjects.

File Name: Supplementary Software 1

Description: Code of runnable demo with corresponding sample dataset and pre-trained model.
