# Supplementary figures and images for: Semantic regularization of electromagnetic inverse problems
Source: Nat Commun. 2024 May 8;15:3869. doi: 10.1038/s41467-024-48115-5 (PMC11079068; doi:10.1038/s41467-024-48115-5)

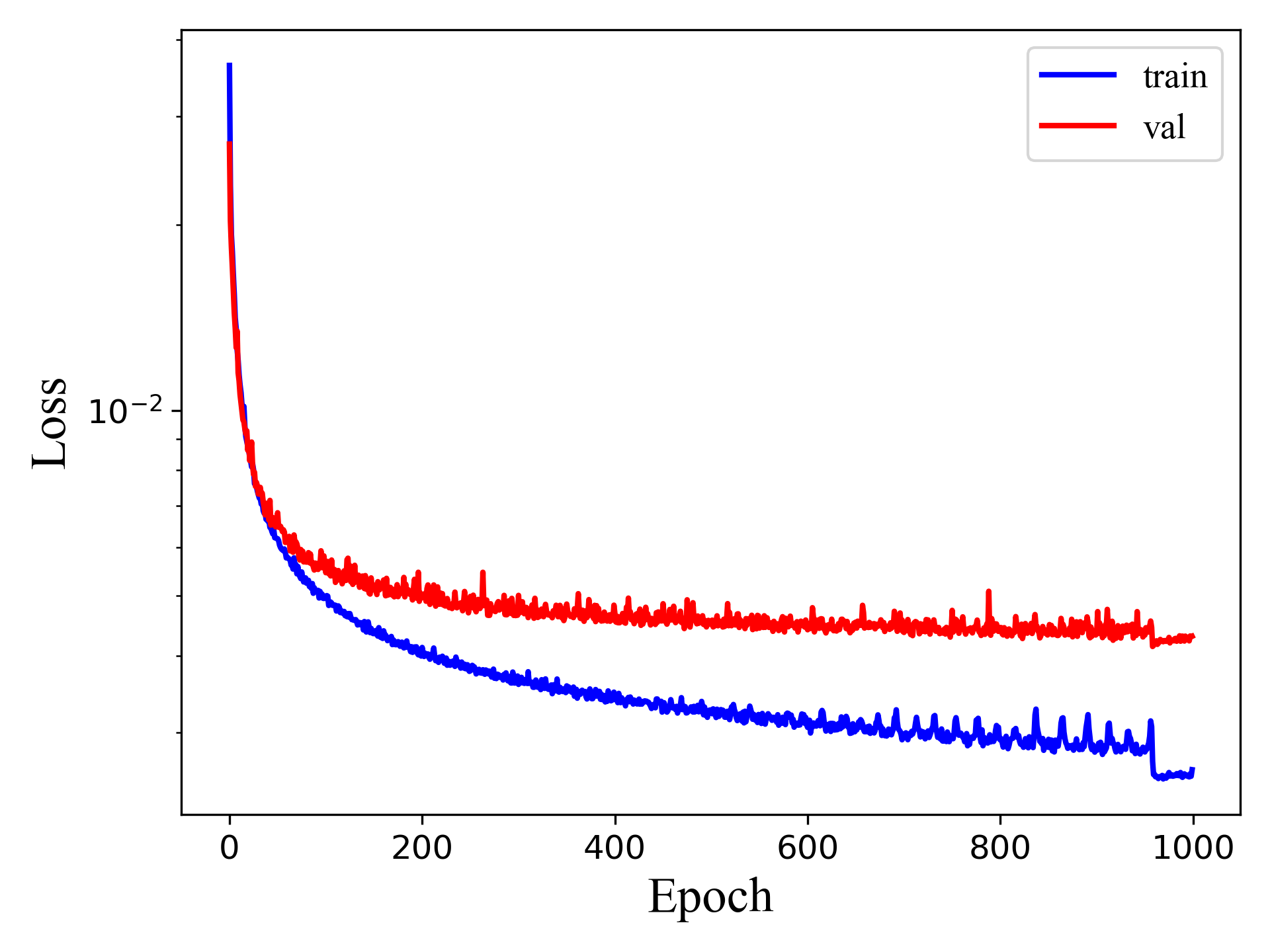

Supplement: Supplementary file 6 — Supplementary Software 1 [file 41467_2024_48115_MOESM6_ESM.zip › demo/checkpoint/Summary/epsr_loss.png]

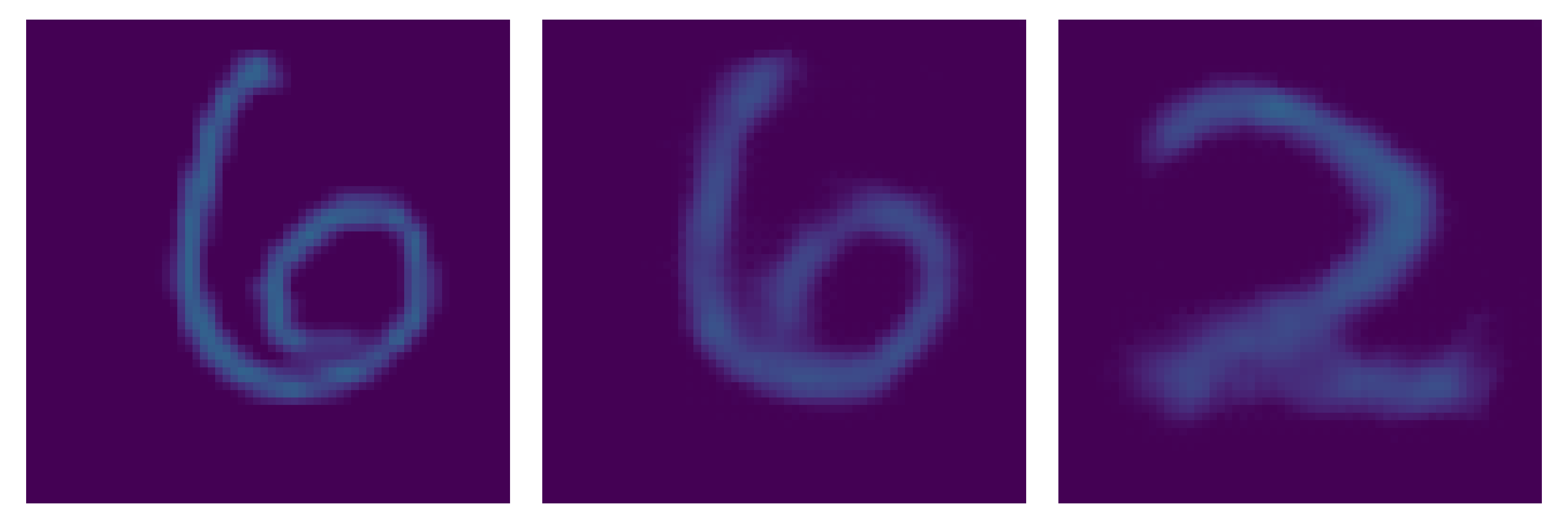

Supplement: Supplementary file 6 — Supplementary Software 1 [file 41467_2024_48115_MOESM6_ESM.zip › demo/figs/100.png]
